# XLVII Reunión Anual de la Sociedad Andaluza de Neurología

**DOI:** 10.31083/RN57192

**Published:** 2026-10-12

## 1. Comunicaciones Orales

**Oral 01**

**Resistencia Primaria a Antiagregantes en el Tratamiento de la Estenosis Carotídea. Serie de Casos**

Manuel García Ortíz^1^, Alejandro Molinero Marcos^1^, Raúl Rashid Abdu Rahim López^1^, Ivana Carolina Micheli^1^, Cecilia Coronado Puerto^1^, Irene Serrano García^1^

^1^Servicio de Neurología, Hospital Universitario Puerta del Mar, Cádiz, España

**Objetivos:** Serie de casos de re-estenosis/trombosis intraluminal como complicación asociada al stenting y/o endarterecto-mía carotídea (TEA) secundaria a resistencia primaria a antiagregantes. **Material y Métodos:** Entre 12/2024–05/2025 se realiza control neurosonológico de patología carotídea a pacientes mono o doble-mente antiagregados con AAS y/o Clopidogrel en los que valoramos: - Presencia de agregación plaquetaria/trombosis intraluminal según la clasificación PLAQUE-RADs (4c) pese a adecuada toma de medicación y control de FRV. - Ausencia de factores que aumentan la agregación plaquetaria de manera fisiológica. - Ausencia de fármacos inhibidores del citocromo CYP 450. **Resultados y Conclusiones:** 5 pacientes presentaban re-estenosis tras TEA o stenting, por lo que se solicitó estudio de verificación de disfunción plaquetaria mediante el análisis de los tiempos Aspirin Reaction Units (ARU) y Unidad de Respuesta Plaquetaria (PRU). - 3 pacientes presentaban resistencia primaria a clopidogrel. Ninguno a AAS. - 2 pacientes presentaban adecuada disfunción plaquetaria. En uno de ellos se identificó la toma concomitante de AINES, en el otro se desconoce factor. Clopidogrel requiere del citocromo para alcanzar su forma activa y en pacientes con isoformas de metabolización lenta, no se llegan a alcanzar niveles suficientes con el consecuente aumento del riesgo de trombosis. Los AINES sobresaturan la enzima COX y dificultan la disfunción plaquetaria asociada a AAS. El 20% de la población puede ser resistente a la disfunción plaquetaria por AAS y hasta el 5–10% por Clopidogrel. Esto debe ser tenido en cuenta en la evaluación de pacientes con re-estenosis carotídea, dado que existen alternativas como Ticagrelor que no requieren metabolización previa (PEGASUS).

**Oral 02**

**Mejora Diagnóstica en Enfermedad de Alzheimer Gracias a la PET de Amiloide: 10 Años de Experiencia en Nuestro Centro**

Amelia Garre Lerma^1^, Marta Molina Haro^1^, Fátima Peral Dorado^1^, Juan Carlos Romero Fábrega^1^, Adrián Piñeiro Donis^2^, Ismael Carrera Muñoz^1^

^1^Servicio de Neurología, Hospital Universitario Virgen de las Nieves, Granada, España

^2^Servicio de Medicina Nuclear, Hospital Universitario Virgen de las Nieves, Granada, España

**Objetivos:** Evaluar la mejora en la certidumbre diagnóstica para la Enfermedad de Alzheimer (EA) en nuestro centro gracias a la PET de amiloide. **Material y Métodos:** Estudio observacional retrospectivo de todos los pacientes de nuestra unidad con PET amiloide con Florbetabén (PET-FBB) entre mayo de 2015 y marzo de 2025. Se establecieron 7 indicaciones para PET-FBB, y resultado en la prueba normal o patológico, con un grado de certidumbre diagnóstica para EA pre y post PET-FBB dividido en 5 categorías (muy poco probable, poco probable, probabilidad intermedia, muy probable y prácticamente seguro). **Resultados y Conclusiones:** 302 sujetos (52,31% mujeres) con 62,73 ± 6,87 años (media ± de). 102 sujetos (33,77%) disponían de punción lumbar (PL). 140 sujetos (46,36%) presentaron PET-FBB positivo para EA. La PET se realizó en 50 sujetos por contraindicación para PL (16,55%), 51 por imposibilidad de PL (16,88%) y 87 por LCR no concluyente (28,80%). La certidumbre diagnóstica pre y post PET-FBB fue: Muy poco probable 2 sujetos (0,66%) y 153 su-jetos (50,66%) respectivamente, poco probable 58 (19,20%) y 0 (0%), probabilidad intermedia 161 (53,35%) y 2 (0,66%), muy probable 63 (20,86%) y 5 (1,65%), y prácticamente seguro 18 (5,96%) y 142 (47,01%). Al agrupar las categorías en solo 2 (alta o baja certidumbre diagnóstica), 20 sujetos (6,62%) fueron catalogados con alta certidumbre diagnóstica pre-PET frente a 295 (97,7%) post-PET (*p* < 0,001). En nuestro centro la PET-FBB permite mejorar de forma muy significativa el grado de certidumbre diagnóstica para la EA en pacientes correctamente seleccionados, especialmente aquellos con imposibilidad para PL o LCR no concluyente.

**Oral 03**

**Respuesta a Triptanes en Pacientes con Migraña Crónica Refractaria**

Laura Ontiveros Campos^1^, Pablo Olea Rodríguez^1^, Laura Amaya Pascasio^1^, Antonio Arjona Padillo^1^

^1^Servicio de Neurología, Hospital Universitario Torrecárdenas, Almería, España

**Objetivos:** Evaluar la tasa de respuesta a triptanes durante las crisis agudas de migraña, en pacientes con migraña crónica refractaria, y determinar si existen variables clínico-demográficas asociadas a dicha respuesta. **Material y Métodos:** Se realizó un estudio descriptivo trasversal en una muestra de pacientes diagnosticados de migraña crónica refractaria a múltiples tratamientos preventivos (fármacos orales clásicos y toxina botulínica), como parte de un análisis post hoc del estudio RAFEMIG (Ecogenicidad del rafe como marcador pronóstico de respuesta al tratamiento en migraña). Se incluyeron un total de 54 pacientes (46 mujeres y 8 hombres), con edades compren-didas entre 24 y 77 años. La respuesta a triptanes se evaluó mediante un cuestionario sencillo de tres preguntas cerradas, basado en los criterios de consenso de la European Headache Federation (EHF), clasificando a los pacientes en dos grupos: respondedores y no respondedores. Por otro lado, se realizó un análisis estadístico de las variables clínicas y demográficas incluidas en el estudio, fijando un nivel de significación *p* < 0,05. **Resultados y Conclusiones:** El 33,3% de los pacientes (n = 18) se consideró respondedor a triptanes. Además, la tasa de respuesta fue ligeramente superior en mujeres (34,8%), que en hombres (25%). No se encontró una asociación estadísticamente significativa entre ninguna de las variables clínico-demográficas estudiadas. En definitiva, la eficacia de los triptanes en el tratamiento agudo de las crisis de migraña, en nuestra muestra, fue baja. Estos resultados refuerzan la necesidad de desarrollar nuevas estrategias terapéuticas eficaces para los pacientes con migraña crónica refractaria, dado el impacto de esta patología en su calidad de vida.

**Oral 04**

**Impacto Económico de las Nuevas Terapias Para Miastenia Gravis en Andalucía: Estudio Comparativo Directo de Ravulizumab vs Efgartigimod en Vida Real**

Pablo Hernández Vitorique^1^, Minerva Mañez Sierra^1^, Guillermina García Martin^2^, Javier Pinel Ríos^3^, Natalia García Casares^4^, Pilar Carbonell Crovillo^1^

^1^Servicio de Neurología, Hospital Universitario Virgen de la Victoria, Malaga, España

^2^Servicio de Neurología, Hospital Universitario Regional de Málaga, Malaga, España

^3^Servicio de Neurología, Hospital Quirón Salud, Malaga, España

^4^Facultad de Medicina Málaga, Malaga, España

**Objetivos:** Ravulizumab y efgartigimod han ampliado significativamente las opciones terapéuticas para la Miastenia Gravis generalizada (MGg). No obstante, su uso en la práctica clínica puede verse restringido por criterios adicionales a los establecidos en ficha técnica, frecuentemente relacionados con políticas hospitalarias orientadas al control del gasto. Este estudio evalúa el impacto clínico y económico del uso precoz de estas terapias en Andalucía para comparar la efectividad y seguridad de ravulizumab y efgartigimod en condiciones de vida real, y estimar el posible ahorro sanitario derivado de su uso temprano. **Material y Métodos:** Estudio observacional, retrospectivo y multicéntrico. Se incluyeron pacientes adultos con MGg AChR+, refractarios a ≥2 inmunosupresores convencionales, con MG-ADL basal > o igual a 6. Se analizaron variables clínicas, de seguridad y consumo de recursos sanitarios, con un seguimiento mínimo de 6 meses. **Resultados y Conclusiones:** Se incluyeron 13 pacientes (2 con ravulizumab, 9 con efgartigimod y 2 con switch de efgartigimod a ravulizumab). Ambos grupos presentaron mejoría clínica sostenida (MG-ADL, MGFA, MSE), sin efectos adversos graves. Todos los pacientes cumplieron criterios de ficha técnica y requisitos hospitalarios adicionales. Ninguno precisó inmunoglobulinas ni plasmaféresis; solo dos pacientes con efgartigimod requirieron ingreso hospitalario, sin necesidad de UCI. El ahorro medio estimado fue de 6.800 €/paciente en 6 meses (88.500 € en total). Ravulizumab y efgartigimod son tratamientos eficaces, seguros y potencialmente coste-efectivos en MGg. La eliminación de trabas administrativas para su uso precoz podría optimizar resultados clínicos y reducir costes sanitarios innecesarios.

**Oral 05**

**Prevalencia de “Deterioro Cognitivo Ligero” Subyacente en Población con Antecedentes Familiares de Enfermedad de Alzheimer y con “Deterioro Cognitivo Subjetivo-Plus” en la Provincia de Sevilla. Estudio Clínico Observacional Transversal**

Ángeles Barro Crespo^1,2^, José Manuel López Domínguez^1,2^, Félix Viñuela Fernández^1,2^

^1^Unidad de Neurociencias, Hospital Victoria Eugenia, Sevilla, España

^2^Unidad de Deterioro Cognitivo, Servicio de Neurología. Hospital Universitario Virgen Macarena, Sevilla, España

**Objetivos:** Establecer la prevalencia de casos con deterioro cognitivo ligero amnésico (DCL-amnésico) sin detectar en población con Subjective cognitive decline - plus (SCD-plus) a riesgo. Establecer relaciones entre la intensidad de las quejas subjetivas y otras variables con la presencia de deterioro cognitivo objetivo, así como la relación de dichas quejas con variables atencionales. **Material y Métodos:** Estudio clínico observacional transversal no comparativo (46 sujetos sanos, sin diagnóstico confirmado de deterioro cognitivo objetivo, con SCD plus, con ≥60 años). Repeatable Battery for the Assessment of Neuropsychological Status (RBANS versión-A), Subjective Cognitive Decline Questionnaire (SCD-Q), Cuestionario Reserva Cog-nitiva (CRC); Hospital Anxiety and Depression Scale (HADS). **Resultados y Conclusiones:** La prevalencia del DCL-amnésico es 10,87%. No se ha encontrado relación entre el sexo, nivel académico, reserva cognitiva, edad, antecedente primer/segundo grado, factores riesgo vascular, ansiedad o depresión, intensidad de las quejas cognitivas (participante/familiar) y la presencia de deterioro cognitivo. Se establece relación entre la sintomatología depresiva y la presencia de alteraciones atencionales. La ausencia de correlación entre la intensidad de las quejas subjetivas y la presencia de deterioro cognitivo, indica la necesidad de realizar una valoración neuropsicológica multidominio a todos los pacientes con quejas subjetivas, para distinguir dichas quejas de un deterioro cognitivo objetivo y ofrecer tratamientos modificadores de la EA a los que tenga biomarcadores positivos. Estableciendo el punto de corte para DCL en -1DE (criterios DSM-5), se puede diferenciar las quejas subjetivas de un deterioro cognitivo ligero, evitando así falsos negativos.

**Oral 06**

**Vacunación Contra el Virus de la Hepatitis B en la Esclerosis Múltiple Remitente-Recurrente**

Jerónimo Cruces Párraga^1^, Francisco Javier Serrano García^1^, Pablo Doblas Raez^1^, Eduardo Agüera Morales^1^

^1^Servicio de Neurología, Hospital Universitario Reina Sofía, Córdoba, España

**Objetivos:** Analizar la respuesta a la vacuna del virus de la hepatitis B (VHB) en términos de título de Ac (IgG anti-VHB) en pacientes con esclerosis múltiple (EM) que utilizan terapias modificadoras de la enfermedad (TME) como tratamiento. **Material y Métodos:** Estudio de cohortes retrospectivo en pacientes con EM vacunados del VHB. Se determina la inmunidad adquirida tras la vacunación y se realiza un análisis de la inmunogenicidad en función del tipo de TME, dosis de la vacuna, edad y sexo. Se considera buena respuesta a vacunación si el título de IgG anti-VHB es ≥10 mIU/mL. Se aplicó estadística descriptiva e inferencial. Este trabajo dispone de un informe favorable del Comité de Ética de la Investigación. **Resultados y Conclusiones:** Se incluyeron 200 pacientes: 58 varones (29%) y 142 mujeres (71%), con edad media de 47,28 años (IC del 95%: 45,99–48,56). Se observó una menor respuesta a la vacuna en los pacientes tratados con TME inmunosupresoras (69,4%, *p* < 0,001), concretamente, con fingolimod (34,4%, <0,001), aunque mayor con teriflunomida e interferón beta (100%, *p* < 0,001). No se hallaron diferencias entre pacientes que siempre mantuvieron la misma TME frente aquellos que la modificaron durante la vacunación. También se demostró una asociación estadística entre el número de ciclos de vacuna administrados y la respuesta inmune resultante, pero no entre la edad y el género. El nivel de inmunización tras la vacunación frente al VHB en pacientes con EM disminuye debido al uso de TME y se ve influido por el número de ciclos de la vacuna.

**Oral 07**

**Análisis de las Características Basales de la Cohorte Multicéntrica Andaluza Neuro-Reca/Roche (Proyecto Medicina Personalizada; Programa Alzheimer)**

Emilio Franco Macías^1^, Beatriz Espejo Martínez^2^, Rocío Hervás Navidad^2^, Silvia Rodrigo Herrero^3^, Ismael Carrera Muñoz^4^, Begoña Oliver Martos^5^

^1^Servicio de Neurología, Hospital Universitario Virgen del Rocío, Sevilla, España

^2^Servicio de Neurología, Hospital Universitario San Cecilio, Granada, España

^3^Servicio de Neurología, Hospital Universitario Juan Ramón Jiménez, Huelva, España

^4^Servicio de Neurología, Hospital Universitario Virgen de las Nieves, Granada, España

^5^Instituto de Investigación de Biomedicina de Málaga, Málaga, España

**Objetivos:** El objetivo fue analizar las características socio-demográficas, neuropsicológicas y licuorales, a nivel basal, de la cohorte Neuro-Reca/Roche. **Material y Métodos:** En 9 Unidades de Memoria del SSPA, se incluyeron pacientes con sospecha de enfermedad de Alzheimer prodrómica (MMSE ≥22, puntuación total TMA-93 < percentil 10 y CDR-GS = 0,5). Se administró una batería neuropsicológica amplia (cognitiva- FCSRT, Stroop, Digit span, Copia figura simple RBANS, VOSP localización, Figuras superpuestas-; conductual-MBI-C-; funcional-FAQ-) y se realizó estudio de biomarcadores en LCR (medición con métodos automatizados: Elecsys, Lumipulse o Euroimmun). Muestras de plasma y LCR fueron donadas a Biobanco para objetivos posteriores de validación y de predicción de conversión a demencia. **Resultados y Conclusiones:** Resultados: 120 pacientes incluidos. Variables cuantitativas expresadas en mediana y (rango intercuartílico). Edad, 71 (65–77) años; 45,8%, mujeres; 18,3% < estudios primarios; cognición global, MMSE, 24 (23–27). 71 pacientes (59,2%), biomarcadores positivos; 49 pacientes (40,8%), biomarcadores negativos. El grupo Alzheimer tuvo puntuaciones más bajas en memoria: FCSRT verbal: Recuerdo Libre [7 (5–12) vs 12 (7–18), *p* < 0,001]; Recuerdo Total [22 (13–33) vs 31 (21–39), *p* < 0,01]. El grupo no Alzheimer tuvo puntuaciones más alta en MBI-C: [16 (8–23] vs 8 (3–14), *p* < 0,005]. Conclusiones: La ratio Alzheimer/No Alzheimer de la cohorte, 60:40, es óptima para el objetivo de validación de biomarcadores en plasma en nuestra Comunidad. El grupo de 71 pacientes con EA prodrómica servirá para analizar biomarcadores predictores de riesgo de conversión rápida a demencia.

**Oral 08**

**Estudio Histopatológico del Endotelio en el Trombo Obtenido Tras Trombectomía en Fase Aguda del Ictus Isquémico**

Claudio Alejandro López Mesa^1^, Clara Chamorro Santos^2^, Javier Arnedo Fernández^3,4^, Irene Pérez Ortega^1^, María Dolores Fernández Pérez^1^, Juan Carlos Romero Fábrega^1^

^1^Servicio de Neurología, Hospital Universitario Virgen de las Nieves, Granada, España

^2^Servicio de Anatomía Patológica, Hospital Universitario Virgen de las Nieves, Granada, España

^3^Departamento de Estadística e Investigación Operativa, Universidad de Granada, Granada, España

^4^Instituto de Investigación Biosanitaria IBS Granada, Granada, España

**Objetivos:** Evaluar la utilidad diagnóstica de analizar la presencia de células endoteliales en el trombo obtenido mediante trombectomía en la fase aguda del ictus isquémico. **Material y Métodos:** Estudio prospectivo de pacientes consecutivos con ictus isquémico tratado mediante trombectomía en fase aguda. Los trombos se fijaron en parafina y se realizaron cortes al microtomo. Para identificar las células endoteliales se utilizó la tinción inmunohistoquímica CD34. Una anatomopatóloga experta examinó cada muestra de forma cualitativa, determinando la presencia o ausencia de endotelio. Finalmente, se analizó la asociación entre la presencia de endotelio y la etiología del ictus, según la clasificación TOAST. **Resultados y Conclusiones:** Se analizaron los cortes histopatológicos de 200 pacientes. Se observaron diferencias significativas en cuanto a la presencia de endotelio según la etiología del ictus. La presencia de endotelio fue significativamente mayor en trombos aterotrombóticos (42,3%) que cardioembólicos (11,1%), criptogénicos (10,6%) y otros (20,0%) [*p* < 0,001]. La presencia de endotelio también se asoció de forma significativa con tener antecedentes de enfermedad cardiovascular [*p* = 0,008] y con la realización de angioplastia durante el tratamiento endovascular [*p* = 0,014]. Además, se detectó endotelio en un caso aislado con etiología infrecuente por web carotideo. La presencia de células endoteliales en el trombo obtenido mediante trombectomía mecánica en fase aguda del ictus isquémico se asocia con la etiología aterotrombótica, y puede ser una herramienta útil para orientar el diagnóstico en casos de origen indeterminado. Hasta donde alcanza nuestra revisión, no hemos encontrado precedentes en la literatura científica que relacionen la detección de endotelio en el trombo con la etiología del ictus.

## 2. Comunicaciones Póster

**Póster 01**

**Kabuki y Devic: ¿Un Nuevo Comienzo?**

Marta Molina Haro^1^, José María Barrios López^1^, María Del Carmen Barrera Aguilar^1^, Miguel Ángel López Nevot^1^

^1^Servicio de Neurología, Hospital Universitario Virgen de las Nieves, Granada, España

**Objetivos:** Describir el caso de un paciente con diagnóstico de trastorno del espectro de la neuromielitis óptica (TENMO) y un síndrome genético. Revisamos su posible vinculación inmunológica e implicaciones terapéuticas. **Material y Métodos:** Varón de 17 años con malformaciones (atresia coanal, agenesia lacrimal), hipotiroidismo, hipoacusia e infecciones recurrentes. Ingresa por pérdida visual subaguda, con hallazgos clínicos y paraclínicos compatibles con neuritis óptica retrobulbar derecha. Se detectan anticuerpos anti-AQP4 positivos y se diagnóstica de TENMO, tratándose con metilprednisolona intravenosa, seguido de plasmaféresis ante la ausencia de mejoría. Posteriormente se inicia rituximab y se solicita estudio de inmunodeficiencias por los antecedentes infecciosos. Durante el primer año de seguimiento no presenta nueva clínica ni infecciones. Se descartan inmunodeficiencias adquiridas y se detecta una variante probablemente patogénica en el gen KMT2D, compatible con el síndrome de Kabuki tipo 1 y el Síndrome de Anomalías del Arco Braquial. **Resultados y Conclusiones:** El síndrome de Kabuki se caracteriza por anomalías del desarrollo y disfunción inmunológica, asociando enfermedades autoinmunes (como lupus o artritis reumatoide) y predisposición a infecciones. Aunque no hay casos reportados con TENMO, existe una alteración epigenética del factor FOXP3, afectando a los linfocitos T reguladores, lo que sugiere una posible vinculación inmunológica. El tratamiento con rituximab fue seguro, si bien el síndrome de Kabuki asocia alteraciones en linfocitos B e inmunidad humoral. Por ello se plantean alternativas terapéuticas con otras dianas inmunológicas como el eculizumab (complemento) o sartralizumab (interleucina-6). Presentamos el primer caso de TENMO y síndrome de Kabuki. Su coexistencia podría sugerir vinculación inmunológica, con posibles implicaciones fisiopatológicas y terapéuticas.

**Póster 02**

**Temblor Distónico de Manos Tratado Mediante ECP del GPi Bilateral**

Fátima Peral Dorado^1^, Javier Capaje Mosquera^1^, Marta Molina Haro^1^, Raúl Rashid Abdu RahimLópez^2^, Benjamín Iañez Velasco^3^, Francisco Escamilla Sevilla^1,4^

^1^Servicio de Neurología, Hospital Universitario Virgen de las Nieves, Granada, España

^2^Servicio de Neurología, Hospital Universitario Puerta del Ma, Cádiz, España

^3^Servicio de Neurocirugía, Hospital Universitario Virgen de las Nieves, Granada, España

^4^Instituto de Investigación Biosanitaria de Granada (IBS GRANADA), Granada, España

**Objetivos:** En la literatura sobre ECP existe controversia sobre la diana a elegir en temblores distónicos. Presentamos un caso intervenido mediante ECP del GPi bilateral y revisamos la literatura sobre las mejores dianas en casos de temblores distónicos, así como la adecuación de una diana u otra en función de las características clínicas. **Material y Métodos:** Varón de 39 años con hipoxia perinatal y retraso psicomotor leve durante la infancia que comienza a los 12 años de edad con un temblor distónico progresivo de manos, primero la izquierda y años después la derecha junto a la aparición de otros rasgos distónicos en grado leve (torticaput derecho y laterocollis izquierdo). Tuvo un curso relativamente estático empeorando desde los 30 años de edad con una creciente discapacidad por las posturas distónica y el temblor de manos, con refractariedad a los distintos fármacos (levodopa, tetrabenazina, trihexifenidilo, ácido valproico, carbamazepina y toxina botulínica) por lo que se indicó tratamiento mediante ECP del GPi bilateral con importante beneficio clínico y recuperación de autonomía tras dos años de seguimiento postquirúrgico. **Resultados y Conclusiones:** Aunque la ECP es una estrategia consolidada en el tratamiento del temblor distónico no siempre tenemos certeza acerca de cuál es la mejor diana para determinados pacientes. En nuestro paciente elegimos la ECP del GPi bilateral porque le dimos más importancia al componente distónico que el tremórico de manos.

**Póster 03**

**Serie de Casos de Síndrome de Miller–Fisher en 2024 en un Hospital de Tercer Nivel**

Adelaida Cuenca Relinque^1^, Joaquin Arzalluz Luque^1^, Marta Calle Serrano^1^, Fernando Francisco Ortega Ortega^1^, Elena Guijarro Albaladejo^1^

^1^Servicio de Neurología, Hospital Universitario Virgen Macarena, Sevilla, España

**Objetivos:** Analizar las características clínicas, pruebas complementarias, tratamiento y pronóstico de una serie de pacientes con Síndrome de Miller-Fisher. **Material y Métodos:** Estudio descriptivo retrospectivo de serie de casos entre enero-abril 2024. Se incluyeron tres varones con edad mediana 48 años (rango: 25–62). Dos presentaron cuadro infeccioso previo. Dos debutaron con diplopia seguida de inestabilidad y uno con inestabilidad seguida de diplopia. A la exploración todos presentaron oftalmoparesia compleja, arreflexia y ataxia de predominio cerebeloso. Dos asociaron mononeuropatía craneal múltiple con afectación del V motor, VII, IX, X pares y uno asoció paraparesia proximal. **Resultados y Conclusiones:** Un paciente presentó disociación albuminocitológica en líquido cefalorraquídeo, siendo en el resto normal. El estudio neurofisiológico mostró respectivamente disminución de amplitudes de potenciales de acción sensitivos, normalidad en el estudio y ausencia de respuesta H con ondas F normales. Positividad en suero de anticuerpos anti-GQ1b fue 100%. Resto de estudio de causas secundarias metabólicas/tóxicas, autoinmunes, infecciosas o infiltrativas fue normal. Los tres recibieron tratamiento con inmunoglobulinas intravenosas (IgIV) y dos precisaron posteriormente plasmaféresis por déficit discapacitante persistente pese a IgIV. La evolución neurológica fue favorable, presentando al alta hospitalaria el 100% deambulación autónoma con leve ataxia, persistiendo oftalmoparesia. En nuestra serie el 100% presentaron la triada clínica típica y la diplopia fue el síntoma inicial predominante. Las pruebas complementarias mostraron hallazgos sutiles, el diagnóstico de sospecha fue clínico y el tratamiento empírico hasta positividad de anti-GQ1b. La evolución funcional fue favorable con escasa discapacidad al alta hospitalaria: leve ataxia y oftalmoparesia residual.

**Póster 04**

**Estimulación Cerebral Profunda del Globo Pálido Interno (ECP-GPi) Para Control de la Distonía en Neurodegeneración Asociada al Déficit de Pantotenato Kinasa (PKAN): Resultados en dos Hermanos**

Carlota Villar Rodriguez^1^, Diego Villagrán Sancho^1^, Antonio Cristóbal Luque Ambrosiani^2,3^, María Oliver Romero^3,4,5^, Yamin Chocron Gonzalez^4,5^, Fátima Carrillo García^3,4,6^

^1^Servicio de Neurología, Hospital Universitario Virgen del Rocío, Sevilla, España

^2^Unidad de Trastornos del Movimiento, Sevilla, España

^3^Facultad de Medicina, Sevilla, España

^4^Departamento de Neurocirugía, Sevilla, España

^5^Instituto de Biomedicina de Sevilla, Sevilla, España

^6^Servicio de Neurología, Sevilla, España

**Objetivos:** Describir los resultados de ECP-GPi en pacientes mutación en PANK2 en nuestro centro. **Material y Métodos:** Presentamos el caso de dos hermanos portadores de mutación en homocigosis T528M en PANK2 y signo del ojo de tigre en RM. **Resultados y Conclusiones:** CASO-1. Varón de 31 años, inicio de la sintomatología a los 24 años con dificultad en la articulación del lengua-je. Posterior progresión con aparición de distonía generalizada con afectación oromandibular y espasticidad a pesar de tratamiento sintomático. Se realiza ECP-GPi a los 32 años con mejoría de la distonía logrando mayor autonomía y calidad de vida (puntuación Fahn-Mardsen-dystonia-scale-quality-of-life: 21 pre-cirugía–>10 al año post-cirugía). Los efectos de la ECP se mantuvieron, aunque a los 18 meses aparecieron síntomas cognitivos-psiquiátricos típicos de la enfermedad. CASO-2. Varón de 27 años, inicio a los 22 años en forma de alteración de la marcha y disartria. Progresión hasta anartria y caídas diarias, asociando blefaroesmpasmo, distonía generalizada con componente oromandibular y disfagia. Se realiza ECP-GPi a los 28 años. Buena evolución posterior con mejoría de la marcha, disminución de las caídas, de la distonía cervical y del lenguaje que mantiene hasta última revisión (cuatro meses post-intervención). La ECP es eficaz para mejorar la funcionalidad y calidad de vida en pacientes con PKAN y distonía severa. Aunque, la propia progresión de la neurodegeneración, la aparición de síntomas cognitivos y neuropsiquiátricos, así como la heterogeneidad clínica y genética de esta entidad (incluso entre hermanos con la misma mutación), hacen que la respuesta sea menos predecible que en otros casos de distonía idiopática.

**Póster 05**

**La Melanina Salival y la Tirosinasa Sérica se Correlacionan de Modo Inverso con la Duración de la Enfermedad de Parkinson Idiopática**

Emilio Fernández Espejo^1^, Fátima Damas Hermoso^2^

^1^Laboratorio de Medicina Regenerativa, Málaga, España

^2^Servicio de Neurología, Hospital de Valme, Sevilla, España

**Objetivos:** Es conocida la importancia de la neuromelanina en la enfermedad de Parkinson idiopática (EPI), pero sabe muy poco acerca del papel de la melanina no neural en la enfermedad de Parkinson idiopática. Teniendo en consideración que la tirosinasa humana es la enzima principal requerida para la síntesis de melanina, se han analizado las relaciones entre la melanina no neural y la tirosinasa humana en suero y saliva, así como su relación con las características clínicas en la EPI. **Material y Métodos:** La concentración sérica y salival de melanina y tirosinasa se midió mediante ELISA en 50 pacientes y 30 con-troles, y se analizaron las correlaciones con parámetros clínicos. Se valoraron escalas motoras y el régimen de medicación. Hay informe favorable del CEI del Hospital Valme y PEIBA. **Resultados y Conclusiones:** No se han detectado cambios respecto a controles en los niveles de melanina y tirosinasa humana en suero y saliva de los enfermos de Parkinson idiopático. Por tanto, no son biomarcadores válidos de la enfermedad de Parkinson. Sin embargo, la melanina salival y la tirosinasa sérica se correlacionan de modo inverso con la duración de la EPI. Los niveles de melanina y tirosinasa humana en suero y saliva no están correlacionados entre sí. Como conclusión, los niveles de melanina salival y la tirosinasa humana en suero se reducen con el progreso de la EPI, lo que sugiere que podrían ser indicadores periféricos para valorar la duración de la enfermedad.

**Póster 06**

**Takotsubo e Ictus ¿Causa o Consecuencia?**

Macarena González Campos^1^, Gonzalo Torres Sánchez^1^, Hayar El Mouhajir Mohamed^1^

^1^Servicio de Neurología, Hospital Juan Ramón Jiménez, Huelva, España

**Objetivos:** El objetivo principal es explorar la relación bidireccional entre el síndrome de Takotsubo y el ictus, presentando un caso clínico real y realizando una revisión sistemática de la literatura. **Material y Métodos:** Se describe un caso clínico de una mujer de 62 años que tuvo ictus y takotsubo. Se realiza una revisión sistemática mediante la metodología PRISMA incluyendo los artículos en inglés y español acerca del ictus y el takotsubo. **Resultados y Conclusiones:** El caso trata de una mujer de 62 años que inicia de forma brusca con afasia, hemianopsia homónima derecha, desviación oculocefálica, parálisis facial, hemiplejía y hemihipoalgesia derechas. Se realizaron angioTC de TSA, TC craneal, ECG y analítica evidenciándose en estos últimos una elevación del segmento ST y de la troponina T por lo que se realizaron un ecocardiograma y un cateterismo de forma urgen-te, donde se apreciaba discinesia apical con arterias coronarias normales, patrón compatible con Síndrome de Takotsubo. Se inició tratamiento antiagregante y se desestimó tratamiento de reperfusión del ictus por haberse administrado durante la realización del cateterismo heparina. El síndrome de Takotsubo puede actuar tanto como causa como consecuencia de un ictus. Para esclarecer su etiología, resulta fundamental llevar a cabo una historia clínica detallada del paciente. La indicación de anticoagulación está recomendada en presencia de disfunción ventricular o trombo intracardiaco. La relación etiopatogénica sigue siendo un área de investigación en desarrollo, lo que resalta la necesidad de estudios adicionales para comprender mejor su interconexión y optimizar su manejo clínico.

**Póster 07**

**“Fingolimod off: ¿Despertando a la Bestia?”**

Belén Nieto Verdugo^1^, Julia Zapata Sanjuán^1^, Marta Molina Haro^1^, Francisco Romero Cerrillo^2^, Virginia Guillén Martínez^1^

^1^Servicio de Neurología, Hospital Universitario Virgen de las Nieves, Granada, España

^2^Servicio de Radiodiagnóstico, Hospital Universitario Virgen de las Nieves, Granada, España

**Objetivos:** El rebound por suspensión del fingolimod es una exacerbación clínico-radiológica severa y rápida de la actividad de la esclerosis múltiple remitente recurrente (EMRR) tras la interrupción del fármaco, que puede exceder la actividad previa. **Material y Métodos:** Descripción de un caso de rebound por fingolimod con manifestaciones atípicas. **Resultados y Conclusiones:** Mujer de 43 años con antecedentes de EMRR. Se suspendió tratamiento con Fingolimod por falta de eficacia y estaba pendiente de inicio de antiCD20. Acude a Urgencias por migraña con hemianopsia izquierda de <24 horas de evolución. Se activa Código Ictus, con TC multimodal normal. Tras tratamiento, resolución de la cefalea con persistencia de la hemianopsia, por lo que ingresa en Neurología. En la RM se aprecian múltiples lesiones nuevas en fase inflamatoria activa. Recibió tratamiento con bolos de corticoides durante 5 días y posteriormente se inició antiCD20. Como conclusión, fingolimod actúa como modulador del receptor S1P, secuestrando linfocitos en los ganglios linfáticos. Al suspenderlo, se produce una liberación de linfocitos autorreactivos neuro inflamatorios. Ocurre en las 4–12 semanas posteriores a la retirada y se manifiesta con recaídas clínicas severas. Nuestro caso es atípico porque se manifestó con clínica cortical, lo que ampliaba el diagnóstico diferencial, incluyendo causas vasculares y la posibilidad de leucoencefalopatía multifocal progresiva (LMP). En la RM se aprecian múltiples lesiones nuevas desmielinizantes, en fase inflamatoria activa con signo de la vena central, marcador radiológico valioso que ayuda a distinguir la EM de otras enfermedades que afectan la sustancia blanca cerebral.

**Póster 08**

**Medidas Desesperadas Para Carótidas Desesperadas: Bypass Directo en Enfermedad Aterosclerótica Cerebrovascular**

Álvaro Camero Piñatel^1^, Belén Nieto Verdugo^1^, Raquel Lorenzo López^1^, Ana María Jorques Infante^1^, Irene Pérez Ortega^1^

^1^Servicio de Neurología, Hospital Universitario Virgen de las Nieves, Granada, España

**Objetivos:** Presentar un caso de revascularización quirúrgica mediante bypass en paciente con oclusión carotídea y AITs de repetición pese a tratamiento médico optimizado. **Material y Métodos:** Revisión retrospectiva de un caso con soporte bibliográfico. **Resultados y Conclusiones:** Varón de 63 años con FRV y cardiopatía isquémica severa. Ingresa por focalidad transitoria con oclusión de ACID extracraneal y M1 ipsilateral (esta con recanalización espontánea con estenosis severa residual). Dado de alta con doble antiagregación con reingreso posterior por AITS/limb-shaking, con evidencia radiológica de compensación por síndrome de Moya-Moya y ACoA. Se realiza test de reserva hemodinámica mediante Doppler poco concluyente por escasa colaboración y resulta imposible realizar SPECT, decidiendo en comité multidisciplinar realización de bypass ATS-ACM termino-lateral. Inicialmente estable, de forma ambulatoria se detecta hematoma subdural con efecto masa sobre el bypass, suspendiendo clopidogrel, con reingreso posterior por empeoramiento clínico confirmándose mediante arteriografía oclusión del bypass. La evidencia actual aboga en contra de revascularización mediante bypass en enfermedad aterosclerótica, aunque se están realizando estudios para optimizar la selección de pacientes que podrían beneficiarse de este procedimiento. En este paciente la intervención fue considerada por mala compensación hemodinámica con clínica persistente pese a tratamiento médico optimizado. Una selección subóptima por estudio de reserva hemodinámica deficiente, así como la retirada del clopidogrel y presencia de hematoma con efecto masa sobre el bypass pueden haber contribuido al fracaso de la intervención. Este caso apoya la evidencia existente acerca de la indicación del bypass y podría ayudar a la selección de futuros pacientes

**Póster 09**

**Neuropatía Motora Axonal Hereditaria Asociada a Mutación en el gen VWA1**

Ana Campos Villegas^1^, Pilar Carbonell Corvillo^1^, Antonio Javier Guzmán Téllez^1^

^1^Servicio de Neurología, Hospital Universitario Virgen de la Victoria, Málaga, España

**Objetivos:** El gen Von Willebrand factor A domain containing 1 (VWA1), localizado en el cromosoma 1 (1p36.12), codifica la proteína WARP. Se desconoce exactamente su función, pero se sabe que es crucial para la integridad de la matriz extracelular, interacción con proteínas de adhesión, supervivencia y función de neuronas motoras y sus axones. **Material y Métodos:** Describimos el fenotipo clínico, analítico, neurofisiológico, radiológico y genético en un caso clínico. **Resultados y Conclusiones:** Varón de 16 años que presenta dificultad para deambular leve, así como dificultad para practicar deportes desde la infancia. Es derivado a consulta por hiperCkemia (1000–2548 UI/L). La exploración manifestó limitación a la dorsiflexión de ambos pies con retracción aquílea, leve estepaje y atrofia muscular en piernas. El estudio neurofisiológico mostró disminución severa de amplitud en nervios peroneales (dcho>izdo), con actividad espontánea en ambos músculos tibiales anteriores y patrones de reclutamiento neurógenos. La RM de miembros inferiores evidenció mínima infiltración grasa en compartimento posterior de ambas piernas (dcho>izdo). El estudio genético descartó alteraciones en gen SMN1 y 2, pero identificó una variante probablemente pato-génica en VWA1 en heterocigosis compuesta, confirmando herencia autosómica recesiva (AR) en estudio de segregación familiar. Las mutaciones en el gen VWA1 están asociadas a neuropatía motora hereditaria, de inicio en infancia-adolescencia que se hereda habitualmente de forma AR, con casos descritos en homocigosis y heterocigosis compuesta. Debemos pensar en esta entidad, recientemente conocida, cuando nos encontremos con pacientes con debilidad distal de inicio en infancia-adolescencia que asocian hiperCKemia significativa, manifestada también en otros casos publicados.

**Póster 10**

**Cuando la Risa te Paraliza las Piernas**

María Del Carmen García López^1^, María Jesús Rivera Pinna García^1^, Ana Rodríguez Reguera^1^

^1^Servicio de Neurología, Hospital de Jerez de la Frontera, Jerez De La Frontera, España

**Objetivos:** El óxido nitroso (N_2_O) inhalado, conocido como “gas de la risa”, ha experimentado un aumento en su consumo como droga recreativa. Ejerce su efecto tóxico inactivando de forma irreversible el ion cobalto de la vitamina B12, interfiriendo en la síntesis de mielina, provocando daño a nivel central (degeneración combinada subaguda) y periférico (polineuropatía sensitivo-motora, generalmente sensitiva y axonal). No se detecta en el test de tóxicos rutinario disponible en urgencias. **Material y Métodos:** Descripción retrospectiva de dos casos clínicos con afectación neurológica secundaria al empleo de N_2_O inhalado. **Resultados y Conclusiones:** Varón de 27 años, consulta por déficit sensitivo de rápida instauración en extremidades superiores e inferiores llegando a dificultar la deambulación. Bioquímica de LCR normal. RM de columna cervical: mielitis longitudinal extensa C1–C5 con afectación predominante dorsolateral, respetando asta anterior. Inicialmente recibe corticoides, rehistoriando reconoce consumo de óxido nitroso y recibe tratamiento con vit B12. Evolución favorable con mínimas parestesias residuales a los 3 meses. Mujer de 21 años, rasgos anómalos de personalidad. Presenta ataxia sensitiva severa y dolor neuropático en miembros inferiores de curso ascendente. Exploración: afectación de todas las modalidades sensitivas con nivel sensitivo D6, hiporreflexia generalizada e imposibilidad para deambular. No consiente punción lumbar ni estudios de resonancia. Tras constatar antecedente de consumo de N_2_O, recibe tratamiento con vit B12 y pregabalina. Al alta, precisa silla de ruedas. A los tres meses, deambula con una muleta. Evitar la reexposición al óxido nitroso y la rápida suplementación de vitamina B12 (incluso con niveles analíticos iniciales normales) son claves para minimizar las secuelas.

**Póster 11**

**Epilepsia Farmacorresistente de Difícil Diagnóstico. A Propósito de un Caso**

Adelaida Cuenca Relinque^1^, Susana Lugones Diaz^1^, Marta Calle Serrano^1^, Elena Guijarro Albaladejo^1^, Víctor Carmona Bravo^1^, Luminita Dinca Avarvarei^1^

^1^Servicio de Neurología, Hospital Universitario Virgen Macarena, Sevilla, España

**Objetivos:** Presentamos un caso de epilepsia de difícil diagnóstico, de inicio parietal vs insular, dado que las dos entidades son “grandes simuladoras” y pueden presentar una sintomatología dolorosa; el diagnóstico positivo y el diagnóstico diferencial representan un reto dado que en los registros EEG de superficie no detectan alteraciones localizadores y es necesario realizar una monitorización de larga duración (VEEG); en la mayoría de los casos es imprescindible un estudios con electrodos intracraneales. Presentar el caso de un paciente de 15 años, que en los últimos 3 años refiere episodios paroxísticos de dolor lancinante a nivel del glúteo izquierdo de 20–60 segundos de duración asociado a comportamiento hipermotor y signos vegetativos. Exponemos los resultados de los VEEG realizados en este paciente, además de otras pruebas complementarias y el efecto de los fármacos antiepilépticos administrados. **Material y Métodos:** Se han realizado 8 estudios Video-EEG: 3 en vigilias, 3 en privaciones de sueño, 1 de siesta y un video EEG de larga duración, así como RNM craneal y de columna vertebral, estudio PET-TAC cerebral y ENMG de MMII. **Resultados y Conclusiones:** En los estudios Video-EEG se recoge: actividad interictal en forma de ondas agudas rítmicas a 6–7,5 Hz de mediana amplitud en región frontotemporal derecha; la actividad ictal se inicia en región centroparietal derecha (electrodos C4 y T4). El diagnóstico es epilepsia focal de inicio no motor (sensorial) con preservación del nivel de consciencia, en área somatosensorial parietal versus insular dorsal posterior derechos junto con crisis psicógenas no epilépticas asociados.

**Póster 12**

**Paraparesia Espástica Familiar y Nefropatía Asociada: ¿Una Forma Incompleta del Síndrome de Fitzsimmons-Walson-Mellor o una Nueva Entidad Genética?**

Carlos Algar Ramírez^1^, Renata Petruta Dodu^1^, Alejandro Sarmiento Pita^1^, Nadia Bocero Hanan^1^, Sara Sánchez Gamino^1^, Ignacio Rodriguez Lavado^1^

^1^Servicio de Neurología, Hospital Regional Universitario de Málaga, Málaga, España

**Objetivos:** Describir un caso clínico de paraparesia espástica en el contexto de nefropatía familiar no filiada, con estudio genético revelador de una variante no descrita previamente, y valorar su posible relación con síndromes neuro-genéticos raros. **Material y Métodos:** Paciente con trastorno motor progresivo desde la infancia, sin disfunción cognitiva ni auditiva, y antecedentes familiares de nefropatía hereditaria y paraparesia espástica. Se realizó estudio clínico, analítico, de neuroimagen y análisis genético orientado a nefropatías y trastornos motores hereditarios. **Resultados y Conclusiones:** La paciente presentó paraparesia espástica simétrica sin alteraciones sensitivas ni cerebelosas. Mostraba proteinuria persistente con función renal conservada. Las resonancias cerebral y medular fueron normales, salvo hallazgos degenerativos leves. El estudio genético inicial identificó dos variantes de significado incierto en CUBN, relacionadas con proteinuria benigna recesiva. Un análisis posterior reveló una variante en heterocigosis en AARS1 (c.2446C>T), no descrita previamente, con predicción de impacto funcional potencialmente patogénico. Este gen se ha asociado a neuropatías axonales hereditarias dominantes. La historia familiar sugiere una herencia autosómica dominante con afectación combinada renal y neurológica. El cuadro clínico es compatible con una forma incompleta del síndrome de Fitzsimmons-Walson-Mellor. Este caso plantea la sospecha de un síndrome neuro-renal con herencia dominante, compatible con un fenotipo parcial de Fitzsimmons-Walson-Mellor, aunque sin retraso cognitivo ni sordera. El hallazgo en AARS1 podría representar un fenotipo solapado o una nueva entidad genética aún no caracterizada, lo que subraya la necesidad de estudios familiares y un enfoque clínico-genético conjunto en estos síndromes complejos.

**Póster 13**

**Neuritis Óptica en Mujer Joven: Del Enfoque Inflamatorio al Quirúrgico**

Antonio Cristóbal Luque Ambrosiani^1^, Mikel Salgado Irazábal^1^, Luis Fernández Espigares^1^, Diego Villagrán Sancho^1^, Francisco José Hernández Chamorro^1^, María Bernal Sánchez-Arjona^1^

^1^Servicio de Neurología, Hospital Universitario Virgen del Rocío, Sevilla, España

**Objetivos:** Presentar un caso de neuropatía óptica unilateral en una mujer joven con pérdida visual aguda, destacando la importancia de una evaluación exhaustiva y adecuada correlación clínico-radiológica. **Material y Métodos:** Mujer de 35 años que consultó por visión borrosa y dolor periocular derecho con el movimiento ocular, de inicio súbito, sin fiebre ni infecciones recientes. Presentaba cuadrantanopsia inferior derecha, edema de papila y discreta palidez, sin defecto pupilar aferente relativo. La agudeza visual era de 0,9 en OD y 1,0 en OI. La OCT mostró atrofia de fibras nasales y superiores en OD, y la campimetría reveló defecto arciforme inferior derecho. La RM evidenció un quiste aracnoideo temporal derecho con efecto masa sobre el lóbulo temporal y desplaza-miento del nervio óptico, así como ingurgitación de la vena oftálmica superior derecha. Analíticas y estudio de LCR fueron normales, con BOC negativas. Se trató con corticoides intravenosos, sin mejoría clínica, por lo que se descartó origen desmielinizante y se atribuyó a compresión del quiste. **Resultados y Conclusiones:** Se decidió intervención quirúrgica tras la cual se observó mejoría inmediata de la visión, estabilización de la campimetría y desaparición del dolor. Tras 6 meses, se mantuvo agudeza visual OD 0,9, papila pálida sin progresión, y defecto campimétrico inferior estable. Este caso subraya que los quistes aracnoideos, habitualmente considerados hallazgos incidentales, pueden ser clínicamente relevantes cuando existe correlación anatómica y funcional. La presencia de signos de compresión en neuroimagen, como el desplazamiento del nervio óptico o la ingurgitación venosa, debe motivar la consideración de tratamiento quirúrgico, especialmente tras descartar otras etiologías.

**Póster 14**

**Patrón “Eye of The Tiger” en Resonancia Cerebral: Implicaciones en el Abordaje del Parkinsonismo**

Antonio Cristóbal Luque Ambrosiani^1,2^, Astrid Daniela Adarmes Gómez^3,4^, Laura Muñoz Delgado^2,5^, Elena Ojeda Lepe^4,6^, Silvia Jesús Maestre^1,2^, Pablo Mir Rivera^3,4,7^

^1^Unidad de Trastornos del Movimiento, Sevilla, España

^2^Centro de Investigación Biomédica en Red sobre Enfermedades Neurodegenerativas (CIBERNED), Madrid, España

^3^Servicio de Neurología, Sevilla, España

^4^Instituto de Salud Carlos III, Madrid, España

^5^Instituto de Biomedicina de Sevilla, Sevilla, España

^6^Servicio de Neurología, Hospital Universitario Virgen del Rocío/CSIC/Universidad de Sevilla, Sevilla, España

^7^Departamento de Medicina, Sevilla, España

**Objetivos:** Describir el abordaje de un paciente con parkinsonismo asimétrico y hallazgos sugestivos de “eye of the tiger” en resonancia magnética (RM) cerebral con sospecha de neurodegeneración con acúmulo de hierro cerebral (NBIA). **Material y Métodos:** Varón de 56 años, sin antecedentes familiares ni consanguineidad, que presenta con síndrome rígido-acinético de predominio izquierdo sin signos de atipicidad, con degeneración nigroestriatal asociada y con respuesta a terapia dopaminérgica. En la RM cerebral se identificaron, en T2, hiperintensidades simétricas en la porción central de ambos glóbulos pálidos internos, hipointensos en T1. En T2* se objetivaron hipointensidades puntiformes, especialmente en el lado izquierdo. La secuencia en gradiente SWAN mostró hipointensidades bilaterales y simétricas, compatibles con depósitos de hierro. El TC no mostró calcificaciones en los ganglios basales ni en otras regiones. Se solicitó estudio genético de genes asociados tanto a NBIA como a enfermedad de Parkinson hereditaria. **Resultados y Conclusiones:** El análisis genético no identificó variantes patogénicas. El cuadro clínico y la evolución son compatibles con enfermedad de Parkinson idiopática, con buena respuesta al tratamiento dopaminérgico y sin datos de atipicidad. El hallazgo de un patrón radiológico compatible con “eye of the tiger” vinculado a NBIA, no es patognomónico y puede observarse en otras condiciones, sujetos sanos o expuestos crónicamente a ambientes con potencial carga metálica. En este caso, la ocupación previa como mecánico podría representar un factor contribuyente. El abordaje desde el punto de vista genético es recomendable para descartar casos hereditarios y la interpretación de los hallazgos radiológicos en el contexto clínico es clave para evitar sobrediagnósticos.

**Póster 15**

**Ictus Sin Lateralización Y Neuroimagen Urgente No Concluyente: Cuando La Clínica Guía El Tratamiento**

Antonio Cristóbal Luque Ambrosiani^1^, Manuel Medina Rodríguez^1^, Javier Ciriero Macías^1^, Luis Stalin Manzano Hernández^1^, Pablo Baena Palomino^1^, Leire Ainz Gómez^1^

^1^Servicio de Neurología, Hospital Universitario Virgen del Rocío, Sevilla, España

**Objetivos:** Presentar un caso donde la sospecha clínica fue clave para indicar una arteriografía urgente ante una presentación neurológica grave sin hallazgos concluyentes en la neuroimagen inicial, permitiendo mejorar de forma radical el pronóstico funcional del paciente. **Material y Métodos:** Varón de 62 años, hipertenso sin otros antecedentes. Comenzó bruscamente con estupor, anartria y paraplejía con respuesta cutáneo-plantar extensora bilateral, progresando rápidamente a coma, con ausencia de respuesta a estímulos y mirada fija con pupilas isocóricas reactivas. La exploración orientaba a una afectación mesencefálica o diencefálica bilateral. La TC inicial fue normal, sin apreciar oclusión o estenosis significativa de grandes vasos en angioTC. Pese a ello, dada la presentación y severidad del cuadro clínico, se decidió de forma consensuada con Neurorradiología Intervencionista la realización de una arteriografía urgente ante la sospecha de una oclusión del top basilar. Durante el procedimiento se confirmó dicha oclusión con afectación de la arteria de Percheron, y se realizó trombectomía por aspiración, con recanalización completa e inmediata. **Resultados y Conclusiones:** El paciente pasó rápidamente de un coma profundo a una recuperación progresiva del nivel de consciencia. A los seis meses, era capaz de caminar con ayuda y de realizar actividades básicas de forma autónoma. La arteriografía cerebral ayuda a determinar la presencia de oclusiones vasculares subsidiarias de tratamiento ante elevada sospecha clínica de ictus, aun cuando el angioTC resulta inconcluyente. Su realización de forma precoz es crucial, como en nuestro caso, donde fue determinante para salvar la vida y preservar la funcionalidad del paciente.

**Póster 16**

**Degeneración Combinada Subaguda de la Médula: Causa Atípica de una Entidad Infrecuente**

Ignacio Lopera Rodríguez^1^, Pedro Franco Perejón^1^, Javier Ciriero Macías^1^, Antonio Cristóbal Luque Ambrosiani^1^, Francisco Javier Gómez Fernández^1^

^1^Servicio de Neurología, Hospital Universitario Virgen del Rocío, Sevilla, España

**Objetivos:** Describir un caso de degeneración combinada subaguda de la médula (DCSM) en una paciente con único antecedente de hábito tabáquico que, tras ampliar el diagnóstico diferencial que presentamos, reconoció haber consumido óxido nitroso. **Material y Métodos:** Mujer de 23 años con antecedente previo. Acude en 2024 por acorchamiento ascendente de miembros inferiores e inestabilidad de la marcha progresiva de un mes de evolución, con dificultad para la deambulación y acroparestesias. La exploración reveló una apalestesia hasta arcos costales con nivel sensitivo torácico e hiporreflexia generalizada. Se realizó estudio con analítica incluyendo perfil inmunológico sistémico, cobre y vitaminas del complejo B, análisis de líquido cefalorraquídeo (LCR) y resonancia magnética (RM) de neuroeje para el estudio completo del diagnóstico diferencial. **Resultados y Conclusiones:** Hallazgo aislado en RM de lesión cervical longitudinalmente extensa e hiperintensa en FLAIR y STIR, con imagen de “V” invertida en cordones posteriores, compatible con DCSM. En el resto del estudio se recoge una marcada hipovitaminosis B12 con hiperhomocisteinemia, anemia macrocítica y fólico en rango. Inició tratamiento con cianocobalamina intramuscular y, tras excluir causas sistémicas potencialmente responsables del déficit, la paciente admitió consumir óxido nitroso. Este caso refleja la importancia de incluir en el diagnóstico diferencial del síndrome cordonal posterior una causa atípica tóxico-metabólica como el consumo de óxido nitroso, siendo imprescindible excluir otras causas median-te un estudio completo de LCR y neuroeje por RM como despistaje de patologías más frecuentes asociadas a la DCSM, incluyendo las enfermedades desmielinizantes e infecciones como la sífilis o VIH.

**Póster 17**

**Left Atrial Septal Pouch: ¿Un Nuevo Factor de Riesgo en el Ictus Criptogénico Juvenil?**

Hayar El Mouhajir Mohamed^1^, Gonzalo Torres Sánchez^1^, Macarena González Campos^1^, Jose María Fernández Velasco^1^

^1^Servicio de Neurología, Hospital Juan Ramon Jiménez, Huelva, España

**Objetivos:** El ictus criptogénico representa un desafío diagnóstico, hasta un 25% de casos presentan origen cardioembólico. El left atrial septal pouch (LASP) es una variante anatómica que resulta de la fusión incompleta entre el septum primum y el septum secundum, formando un receso en la aurícula izquierda sin foramen oval permeable. Se ha sugerido su papel en la estasis sanguínea y el embolismo, pero su implicación clínica aún no está bien establecida. **Material y Métodos:** Se describe dos casos de pacientes adolescentes sin patología previas tratados en el hospital Juan Ramón Jiménez con diagnóstico de ictus criptogénico que presentan como hallazgo en la ecografía transesofágica de LASP. **Resultados y Conclusiones:** Una paciente mujer de 13 años presentó hemiplejía izquierda de inicio súbito, mientras que un paciente varón de 14 años debutó como una crisis de desconexión del medio, dado que ambos cuadros se autolimitaron no precisaron tratamiento emergente. La RMN mostró isquemia en el territorio de la arteria cerebral media derecha en ambos casos. Se realizaron estudios autoinmunes, genéticos, de trombofilia y cardiológicos sin hallazgos relevantes. Sin embargo, la ecocardiografía transesofágica detectó la presencia de LASP en ambos pacientes sin FOP asociado. No se documentaron recurrencias durante el seguimiento y, dado que no existe indicación clara en la literatura, no recibieron tratamiento específico. El LASP podría ser un factor de riesgo subestimado en el ictus criptogénico, por lo que su detección debería considerarse en la evaluación etiológica. Su implicación en la fisiopatología del ictus aún requiere mayor estudio para definir su impacto clínico y posibles estrategias terapéuticas.

**Póster 18**

**Rituximab Como Alternativa en el Síndrome de Clippers: A Propósito de un Caso**

Fernando Francisco Ortega Ortega^1^, Joaquín Arzalluz Luque^2^, Miriam Ben Yelun Imserser^3^, Guillermo Navarro Mascarell^3^, Sara Cárdenas Cubero^3^, Sara Eichau Madueño^3^

^1^Servicio de Neurología, Hospital Universitario Virgen Macarena, Sevilla, España

^2^Servicio Neurología, Hospital Universitario Virgen Macarena, Sevilla, España

^3^Servicio Neurología, Hospital Universitario Virgen Macarena, Sevilla, España

**Objetivos:** El síndrome chronic lymphocytic inflammation with pontine perivascular enhancement responsive to steroids (CLIPPERS) es un cuadro inflamatorio con afectación predominante de rombencéfalo, de inicio subagudo y curso recidivante. Se caracteriza por lesiones puntiformes o curvilíneas con realce en resonancia magnética (RM) y una respuesta favorable a corticoides. **Material y Métodos:** Caso clínico con seguimiento de 4 años en la Unidad de Esclerosis Múltiple del Hospital Universitario Virgen Macarena con diagnóstico de probable CLIPPERS, tratada con Rituximab. **Resultados y Conclusiones:** Mujer de 30 años, fumadora, acude por paresia oculomotora de VI par y cefalea hemicraneal izquierda, opresiva, con fotofobia y febrícula de inicio subagudo. La RM cerebral mostró alteraciones en mesencéfalo y pedúnculos cerebrales con captación de contraste. El líquido cefalorraquídeo (LCR) presentó leucocitosis mononuclear. BOC, estudio microbiológico negativo y citometría de flujo negativas. Se inició metilprednisolona con buena respuesta y se pautó descenso progresivo de prednisona al alta. En sucesivas suspensiones de corticoides, presentó un episodio de neuritis óptica retrobulbar y un síndrome bulbo-cerebeloso con disartria, inestabilidad, hemiparesia derecha y dismetría izquierda. Nueva RM con extensión lesional a bulbo, protuberancia y capsula interna y captación de contraste. Finalmente, se instauró Rituximab semestral, logrando estabilidad clínica sin nuevas exacerbaciones en tres años. No se realizó biopsia, dejando abierto el diagnóstico definitivo. Rituximab es una opción eficaz en CLIPPERS, especialmente tras varias recaídas al retirar corticoides. La falta de biomarcadores diagnósticos obliga a la biopsia confirmativa, aunque no siempre se realiza. El seguimiento estrecho es esencial, especialmente ante ausencia de confirmación histológica por riesgo de conversión a linfoma.

**Póster 19**

**Carotidinia Como Debut Atípico de Arteritis de Takayasu**

Laura Ontiveros Campos^1^, Helena Hernández Bustos^2^, Antonio Arjona Padillo^1^

^1^Servicio de Neurología, Hospital Universitario Torrecárdenas, Almería, España

^2^Centro de Salud de San Isidro, Níjar, España

**Objetivos:** El objetivo de este caso clínico es mostrar una presentación inusual de la arteritis de Takayasu y la relevancia de la ultrasonografía para su detección. **Material y Métodos:** Se presenta el caso de una mujer de 34 años, con diagnóstico previo de migraña con aura, que asociaba dolor cervical derecho de un año de evolución, destacando la mejoría de este con la administración de corticoides en ciclos cortos. En la exploración física, el dolor se exacerbaba con la palpación. **Resultados y Conclusiones:** Se remitió para realización de estudio neurosonológico, observándose un engrosamiento marcado y concéntrico de la pared de la arteria carótida común derecha, con grosor íntima-media (GIM) de 3,2 mm, pérdida de la delimitación del complejo íntima/media y disminución de la luz arterial, sin aumento de las velocidades de flujo arterial. Ante este hallazgo, se derivó para estudio de arteritis de gran vaso y, tras la realización de pruebas complementarias, se confirmó el diagnóstico de arteritis de Takayasu, con afectación de la arteria carótida común derecha. La paciente fue tratada con corticoides orales, con desaparición del dolor carotídeo y normalización de parámetros inflamatorios. La arteritis de Takayasu, especialmente en sus fases iniciales, puede presentarse con síntomas poco específicos, como la carotidinia, siendo esta una forma de debut poco habitual. La ultrasonografía carotídea, muestra unos hallazgos característicos que, junto con los hallazgos clínicos y analíticos, pueden ser claves para su identificación precoz y abordaje terapéutico oportuno.

**Póster 20**

**Trastorno Confusional y Conductual, Hallazgo de Neoplasia Gástrica y Positividad de Anticuerpos Antirecoverina**

María Begoña Gómez González^1^, Ana Rodríguez Román^1^, Lucía Moreno Vico^1^, Beatriz Rosado Peña^1^, Míriam Sillero Sánchez^1^, Ana Gómez Roldós^1^

^1^Servicio de Neurología, Hospital Universitario Puerto Real, Puerto Real, España

**Objetivos:** Las enfermedades paraneoplásicas constituyen un reto diagnóstico, especialmente en pacientes sin tumor conocido y por el creciente abanico de anticuerpos descubiertos y sus métodos de detección, no siempre de igual sensibilidad. Son los trastornos psiquiátricos, junto con las crisis epilépticas, la manifestación neurológica más frecuente de las encefalitis paraneoplásicas. **Material y Métodos:** Varón de 58 años que durante un mes antes de la hospitalización presentó apatía, y en este contexto, sufre un episodio de confusión mientras conduce, y trastorno conductual y de la marcha al bajar del vehículo, todo lo cual justificaba y minimizaba. En la exploración había inquietud psicomotora, ligera inestabilidad en la marcha y un lenguaje coherente, y en la Analítica urgente, elevación significativa de reactantes de fase aguda. Como Antecedentes, Hipertensión Arterial e insomnio desde hacía unos meses, para lo que se trató con etumina. **Resultados y Conclusiones:** Inicialmente, y ante la sospecha de una encefalitis viral (medio rural, casos meses antes), se administró aciclovir. Practicamos RM craneal (leve atrofia simétrica frontotemporal, sin realces patológicos con el contraste), estudio de Líquido cefalorraquídeo (no significativo), Electroencefalograma (discreto enlentecimiento) y, ante la ausencia de clara etiología, determinación de Anticuerpos antineuronales (negativos salvo antirecoverina) y TAC corporal (neoplasia gástrica con probable invasión esplénica y pancreática y adenopatías de aspecto patológico). El paciente se trasladó a otro hospital y acabaría falleciendo. El resultado positivo de los anticuerpos antirecoverina y la detección de una neoplasia no conocida permitieron establecer el diagnóstico de Encefalitis paraneoplásica con debut psiquiátrico.

**Póster 21**

**Fibrinólisis en el Ictus Isquémico por Endocarditis Infecciosa: ¿Recanalizar o Desenmascarar Embolismos Múltiples?**

Lucía García Granados^1^, Ignacio Lopera Rodríguez^1^, Josefina Andrada Moreno^1^, Javier Ciriero Macías^1^, Francisco Javier Gómez Fernández^1^

^1^Servicio de Neurología, Hospital Universitario Virgen del Rocío, Sevilla, España

**Objetivos:** Describir el riesgo de transformación hemorrágica intracraneal asociado a los embolismos sépticos en el contexto de una endocarditis infecciosa (EI), así como sus implicaciones en la toma de decisiones terapéuticas en el ictus agudo. Se incluye una revisión y comparación con casos similares publicados. **Material y Métodos:** Presentamos el caso de un varón de 69 años, con antecedentes de hipertensión arterial y dislipemia, que acudió a urgencias por un cuadro brusco de afasia y hemiplejía derecha, activándose Código Ictus. **Resultados y Conclusiones:** La neuroimagen inicial mostró un ASPECTS de 6 y oclusión de la arteria cerebral media M2 izquierda. Se inició fibrinólisis intravenosa (alteplasa 82 mg) seguida de trombectomía mecánica, con grado de recanalización Trombolysis in Cerebral Infarction (TICI) 2c y extracción de un trombo blanquecino. En la anamnesis dirigida, el paciente refirió fiebre y escalofríos en los días previos. El hemocultivo y urocultivo fueron positivos para Staphylococcus aureus meticilín sensible. Ante la sospecha de EI, se suspendió la infusión de alteplasa. La tomografía computarizada (TC) de control mostró múltiples focos hemorrágicos intraparenquimatosos y hemorragia subaracnoidea en áreas de isquemia, compatibles con embolismos sépticos y transformación hemorrágica secundaria a la fibrinólisis. La evolución fue tórpida, con rotura valvular e insuficiencia aórtica grave que requirió cirugía, además de múltiples embolismos sépticos esplénicos, renales y prostáticos. Este caso refleja la dificultad del diagnóstico clínico de EI en el contexto hiperagudo del ictus. Aunque algunos estudios sugieren beneficio trombolítico en casos seleccionados, la mayoría alertan sobre el elevado riesgo de complicaciones hemorrágicas graves en ictus isquémicos relacionados con EI.

**Póster 22**

**Hemorragia Subaracnoidea (HSA) Sin Sangre en el LCR: Cuando la Imagen no lo es Todo. A Propósito de un Caso**

Javier Ciriero Macías^1^, Ignacio Lopera Rodríguez^1^, Josefina Andrada Moreno^1^, Pedro Franco Perejón^1^, Manuel Medina Rodríguez^1^

^1^Servicio de Neurología, Hospital Universitario Virgen del Rocío, Sevilla, España

**Objetivos:** El diagnóstico diferencial de la hemorragia subaracnoidea no aneurismática y no traumática puede ser dificulto-so. Presentamos un caso clínico con sospecha inicial de HSA de la convexidad. **Material y Métodos:** Mujer de 26 años en seguimiento por hematología por leucemia promielocítica aguda (LPA), en tratamiento con mercaptopurina y metotrexate (remisión completa desde mayo/2023). Consulta por cefalea hemicránea progresiva refractaria a analgesia de primer escalón y síntomas neurológicos transitorios. **Resultados y Conclusiones:** La TC craneal inicial evidenció focos de HSA bihemisféricos en los surcos de la convexidad. La resonancia magnética (RM) mostró: (1) realce leptomeníngeo supra e infratentorial, (2) cambios de señal en la secuencia de susceptibilidad magnética (SWI) en los surcos, correlacionadas con las imágenes basales de TC, sugestivas de HSA en este contexto clínico. No obstante, el análisis del LCR reveló los siguientes hallazgos: (1) bioquímica: 48 leucocitos (96% mononucleares), 0 hematíes, 47 mg/dL de glucosa y 35,4 mg/dL de proteínas.; (2) citometría de flujo: población compuesta mayoritariamente por promielocitos atípicos (48,8%). El posterior análisis de médula ósea no mostró infiltración significativa. Tras el diagnóstico, la paciente inició tratamiento quimioterápico intratecal (hidrocortisona, citarabina y metotrexato), y dexametasona y citarabina por vía intravenosa. El presente caso clínico reveló que, (1) ante una posible HSA no aneurismática debe plantearse la infiltración leptomeníngea en el diagnóstico diferencial y (2) la infiltración leptomeníngea puede simular una HSA de la convexidad en neuroimagen, puesto que en nuestra paciente el análisis de LCR excluyó la presencia de hematíes, descartando la presencia de sangre en el espacio subaracnoideo.

**Póster 23**

**Neuropatía Óptica Inflamatoria Crónica Recurrente (CRION): A Propósito de un Caso**

Sara Cárdenas Cubero^1^, Joaquín Arzalluz Luque^1^, Miriam Ben-Yelun Insenser^1^, Rocío López Ruiz^1^, Sara Eichau Madueño^1^

^1^Servicio de Neurología, Hospital Universitario Virgen Macarena, Sevilla, España

**Objetivos:** La neuropatía óptica inflamatoria crónica recurrente (CRION) se caracteriza por neuritis óptica recurrente, res-puesta favorable a corticoides y corticodependencia. Su diagnóstico requiere exclusión de enfermedades desmielinizantes y sistémicas y su manejo precisa inmunosupresión para evitar daño visual permanente. **Material y Métodos:** Presentamos un caso de neuritis óptica recurrente con respuesta favorable a corticoterapia y exhaustivo estudio etiológico negativo, sugestivo de CRION. **Resultados y Conclusiones:** Mujer de 46 años que en marzo de 2024 sufre episodio de pérdida de agudeza visual y discromatopsia en ojo izquierdo; en la exploración se evidenció defecto pupilar aferente relativo y edema de papila en funduscopia. Se diagnosticó de neuritis óptica de origen inflamatorio y se trató con bolos de corticoides, con evolución favorable hasta quedar asintomática. Se realizó amplio estudio complementario, destacando RMN normal, líquido cefalorraquídeo sin alteraciones incluyendo bandas oligoclonales negativas, y analíticas sin hallazgos incluyendo an-ti-AQP4 y anti-MOG negativos. Tras un año de estabilidad, la paciente sufre recidiva en forma de neuritis óptica bilateral, reintroduciéndose corticoterapia en bolos y posterior pauta oral, con recuperación clínica completa. La OCT confirmó edema de papila bilateral y la RMN la inflamación de ambos nervios ópticos. Dado el curso recidivante con evolución a afectación bilateral y la excelente respuesta a corticoides, se plantea el diagnóstico de CRION y se inicia rituximab para reducir la corticodependencia y prevenir recaídas. Este caso ilustra el desafío diagnóstico de CRION, destacando la importancia de seguimiento estrecho y el uso de inmunomoduladores para preservar la función visual minimizando la iatrogenia asociada a esteroides.

**Póster 24**

**Cuando la Mirada Desafía la Lógica: ‘Wrong-Way Eyes’ y su Valor en la Práctica Clínica**

Coral Conde Velasco^1^, Alexandra Rincón Valencia^1^, Ignacio Ruiz Salcedo^1^, Víctor Carmona Bravo^1^, Soledad Pérez Sánchez^1^, Joan Montaner Villalonga^1^

^1^Servicio de Neurología, Hospital Virgen Macarena, Sevilla, España

**Objetivos:** Tras un ictus supratentorial isquémico/hemorrágico es común observar una desviación conjugada de la mirada hacia el lado de la lesión por afectación de las vías supranucleares descendentes del control ocular. Sin embargo, en ictus talámicos puede presentarse una desviación ocular contralateral, fenómeno llamado “wrong-way eyes”. Esto obliga a considerar etiologías como la epilepsia en el diagnóstico diferencial, haciendo crucial su conocimiento. **Material y Métodos:** Varón de 83 años, hipertenso, dislipémico, trasladado a urgencias tras ser encontrado con bajo nivel de conciencia. A su llegada somnoliento, con desviación de la mirada hacia la izquierda y déficit sensitivo-motor izquierdo. El TC craneal reveló hemorragia talámica derecha. La desviación ocular contralateral a la lesión planteó inicial-mente un diagnóstico diferencial con crisis epilépticas. Aunque no existía otra semiología sugestiva de origen comicial, se realizó ensayo terapéutico con FACs sin cambios. Durante el ingreso, el EEGv sin actividad epileptiforme y la mejoría progresiva de la focalidad, incluida la desviación, respaldaron la teoría de que la DOC se debía a un “wrong-way eyes” por afectación talámica. **Resultados y Conclusiones:** Aunque la desviación de la mirada conjugada suele dirigirse al lado ipsilateral a la lesión, infrecuentemente ocurre una desviación contralateral, conocida como “wrong-way eyes”, en ictus hemorrágicos (o isquémicos) talámicos. La fisiopatología es incierta, pero se proponen mecanismos como compresión mesencefálica con afectación de fibras frontopontinas cruzadas, efectos irritativos sobre núcleos talámicos intralaminares o desequilibrio interhemisférico del sistema de seguimiento ocular. Comprender este signo es clave para orientar el diagnóstico y manejo, reflejando la relevancia de la semiología en neurología.

**Póster 25**

**“Más Allá del Patrón Simétrico: Diagnóstico de Madsam y Tratamiento Eficaz con Inmunoglobulinas en Dosis Ajustadas”**

Pablo Andrés García Núñez^1^, Antonio Cristóbal Luque-Ambrosiani^1^

^1^Servicio de Neurología, Hospital Universitario Virgen del Rocío, Sevilla, España

**Objetivos:** Describir las características clínicas, electrofisiológicas y la evolución terapéutica de un paciente adulto con neuropatía sensitiva y motora desmielinizante multifocal adquirida (MADSAM), ilustrando los hallazgos que lo diferencian de la CIDP típica. **Material y Métodos:** Varón de 33 años, sin antecedentes neurológicos de interés, que consulta por debilidad asimétrica progresiva en miembros superiores (MRC 3/5) e inferiores (MRC 4/5). Tras mejoría parcial tras ingreso hospitalario, presenta recaída clínica en el seguimiento ambulatorio. Se realizaron estudios neurofisiológicos seriados (ENG) que mostraron bloqueos de conducción motores y sensitivo-motores multifocales (en nervios medianos y cubitales), con latencias prolongadas y velocidades reducidas. El LCR mostró hiperproteinorraquia (0,65 g/L) sin pleocitosis. Los perfiles analíticos inmunológicos con marcadores tumorales y la RM craneomedular fueron normales. **Resultados y Conclusiones:** Se diagnosticó una neuropatía desmielinizante adquirida multifocal y de predominio motor (MADSAM) según criterios EFNS/PNS. Se inició tratamiento con IgIV (2 g/kg) repartidos en tres ciclos entre noviembre 2023 y ene-ro 2024. En seguimiento, y conforme a las guías clínicas, se ajustó la dosis de mantenimiento a 1,5 g/kg cada cinco semanas (superior al estándar de 1 g/kg), en base a la respuesta clínica parcial previa. El paciente mostró recuperación funcional casi completa en miembros superiores y estabilización en inferiores. Este caso refleja el valor del patrón asimétrico y multifocal de los bloqueos de conducción en el diagnóstico de MADSAM, además de la utilidad de los ENG seriados en el seguimiento de esta entidad. La adaptación de la dosis de IgIV a la evolución clínica, dentro del margen recomendado por guías, puede optimizar los resultados funcionales a largo plazo.

**Póster 26**

Hemorragia Cerebral Asociada al uso Crónico de Simpaticomiméticos Nasales: Una Complicación Infravalorada

**Jorge Román Rueda^1^, Ignacio Lopera Rodríguez^1^, Luis Fernández Espigares^1^, Carlota Villar Rodríguez^1^, Antonio Cristóbal Luque Ambrosiani^1^, Andrés Hermosín Gómez^1^**

^1^Servicio de Neurología, Hospital Universitario Virgen del Rocío, Sevilla, España

**Objetivos:** Describir un posible caso de hemorragia cerebral asociada al uso abusivo de simpaticomiméticos nasales. Se busca alertar sobre esta rara pero grave complicación. **Material y Métodos:** Varón de 59 años con antecedentes de dislipemia, obesidad, tabaquismo, apnea del sueño y rinitis, sin hipertensión diagnosticada. Como tratamientos, solo usaba simvastatina y oximetazolina nasal. **Resultados y Conclusiones:** En marzo de 2025 acude por debilidad en hemicuerpo izquierdo de inicio ictal tras aplicación de oximetazolina. NIHSS de 7, cifras tensionales moderadas (TA 160/80 mmHg) y TC con hematoma en ganglios basales derechos. Durante el ingreso, permaneció estable, sin necesidad de antihipertensivos intravenosos. Las cifras tensionales fueron adecuadas con enalapril 5 mg cada 24 h. Se completó estudio con analítica y RM craneal, sin hallazgos que sugieran otro origen. Fue dado de alta con diagnóstico de hemorragia cerebral probablemente asociada al consumo crónico de simpaticomiméticos nasales. Pese a los factores de riesgo vascular conocidos, como dislipemia y obesidad, en nuestro paciente no se registraron cifras tensionales elevadas. Obligando a considerar otros desencadenantes, como el uso abusivo de simpaticomiméticos nasales, que refería emplear de forma habitual. La literatura recoge casos aislados de hemorragias cerebrales asociadas al uso nasal de alfa-adrenérgicos, que pueden atravesar la barrera hematoencefálica y provocar efectos vasopresores o picos hipertensivos. Aunque infrecuente, su gravedad potencial justifica limitar su uso a 3–5 días, emplear alternativas y concienciar sobre su efecto adictivo y los riesgos del acceso sin control médico.

**Póster 27**

**Ravulizumab Como Alternativa Terapéutica en Pacientes Oncológicos con Miastenia Gravis**

Paola Estefanía Pesántez Salinas^1^, Begoña Zapata Macias^1^, Alejandro Molinero Marcos^1^, Cecilia Coronado Puerto^1^, Irene Serrano García^1^, Manuel García Ortíz^1^

^1^Servicio de Neurología, Hospital universitario Puerta del Mar, Cádiz, España

**Objetivos:** En la Miastenia Gravis generalizada (MGg) refractaria con anticuerpos antireceptor de acetilcolina (AChR+), existen nuevas dianas terapéuticas como bloqueadores del receptor Fc neonatal (FcRn) e inhibidores del complemento con adecuada eficacia y seguridad. **Material y Métodos:** Varón de 65 años con antecedentes personales de HTA y diverticulosis. Diagnosticado de MGg en 2022 con AChR+ (MGFA IIIb) y crisis miasténica tras un mes del debut. Se inicia tratamiento con prednisona y Azatioprina sin respuesta clínica suficiente tras meses del tratamiento, se decide cambio de azatioprina por tacrólimus con mejoría clínica inicial. Sin embargo, el paciente desarrolla efectos adversos (temblor) y cuadros de diverticulitis agudas de repetición que motivan a reducción de dosis de tacrolimus. En este contexto se realiza TC abominal que muestra lesión tumoral renal izquierda. Tras intento de termoablación se decide seguimiento radiológico. Debido a la relación entre inmunosupresores clásicos y cáncer, se decide suspensión de tacrólimus y se propone Ravulizumab, iniciándose en enero 2024. Tras 10 días de la dosis de carga, paciente consigue mejoría clínica evidente; manteniéndose en este tiempo en mínima manifestación de síntomas (MM) con estabilidad radiológica de la lesión renal. **Resultados y Conclusiones:** El paradigma actual del tratamiento en MGg AChR+ está cambiando gracias a la aprobación de tratamientos más dirigidos como los inhibidores del complemento (Ravulizumab) y los anti FcRn. Debido a la correlación entre la exposición a inmunosupresores clásicos y el riesgo de cáncer, se deben con-templar estos nuevos fármacos como alternativa terapéutica segura; a la espera de mayor experiencia clínica en estudios de vida real.

**Póster 28**

**Un Giro Inesperado**

Julia Zapata Sanjuán^1^, Belén Nieto Verdugo^1^, Pedro Miguel Casanova Leitao^1^, Carmen Valderrama Martín^1^, Iván Maglio Sladogna Barrios^1^, Pedro Pablo Alcázar Romero^1^

^1^Servicio de Neurología, Hospital Universitario Virgen de las Nieves, Granada, España

**Objetivos:** Presentar un caso de Síndrome de Bow Hunter (SBH), causa de insuficiencia vertebro-basilar (VB) infrecuente, que cursó con una presentación clínica peculiar. **Material y Métodos:** Revisión retrospectiva de un caso de SBH y de la literatura. **Resultados y Conclusiones:** Mujer de 77 años con antecedentes de dislipemia y extabaquismo. Ingresa por tetraparesia brusca asimétrica acompañada de cefalea occipital, sin alteraciones aparentes en TC multimodal inicial. Al día siguiente sufre un empeoramiento y en RMN se objetiva una lesión isquémica medular subaguda a nivel de C3–C4 e ictus occipitales agudos silentes. Tras revisar detenidamente la angioRMN, se confirma la presencia de un osteofito a nivel de C4–C5 que comprime la arteria vertebral (AV) izquierda provocando una estenosis dinámica del 50% con disección asociada. Se decide tratamiento conservador con collarín, rehabilitación y anticoagulación por diagnóstico concomitante de FA, tras lo que ha presentado una evolución favorable y sin recurrencias clínicas en 8 meses. El SBH es una causa rara de insuficiencia VB por compresión dinámica de la AV con la rotación cervical, en especial en la unión C1–C2, siendo los osteofitos la causa más común. Suele presentarse en forma de AITs en territorio VB, y se deben buscar mecanismos tromboembólicos no oclusivos como disección, pseudoaneurisma o trombosis en aquellos casos sin oclusión dinámica demostrada. Su manejo suele ser conservador con inmovilización cervical y anticoagulación/antiagregación, con posibilidad de manejo quirúrgico en casos individuales con alto riesgo de recurrencia. Nuestro caso supone una presentación atípica de SBH como síndrome medular anterior.

**Póster 29**

**Rombencefalitis con Afectación Trigeminal**

Julia Zapata Sanjuán^1^, Belén Nieto Verdugo^1^, Carlos Javier Madrid Navarro^1^, Francisco Romero Cerrillo^1^

^1^Servicio de Neurología, Hospital Universitario Virgen de las Nieves, Granada, España

**Objetivos:** Describir un caso de rombencefalitis con afectación clínica y radiológica del nervio trigémino sin aislamiento microbiológico. **Material y Métodos:** Revisión de un caso de rombencefalitis. **Resultados y Conclusiones:** Mujer de 64 años con antecedentes de tabaquismo, HTA, DM2 y dislipemia. Acude a urgencias por parestesias en hemicara derecha seguido de cefalea, disartria e inestabilidad de 3 días de evolución, asociando fiebre de 39 ºC las últimas 24 horas. A la exploración destacaba oftalmoparesia compleja con nistagmo multidireccional, parálisis facial derecha, hipoestesia hemifacial derecha y ataxia. TAC craneal con contraste inicial normal. El LCR mostró pleocitosis (426 cél/µL; mononucleares 32%), hipoglucorraquia y proteinorraquia, con estudio microbiológico en sangre y LCR negativos. Se inició tratamiento empírico con ceftriaxona y ampicilina. Posterior-mente la RMN craneal con contraste mostró lesiones bulboprotuberanciales con realce alrededor de la raíz del trigémino derecho. Autoinmunidad en sangre normal. Ante la sospecha de rombencefalitis por Listeria se añadió gentamicina y dexametasona completando 21 días, con franca mejoría clínica, normalización del LCR y disminución de las lesiones. El aislamiento microbiológico de Listeria monocytogenes es complicado, ya que los cultivos en sangre y LCR son positivos solo en un 61% y 11–41%, respectivamente. En este caso, la neuroimagen característica y la res-puesta favorable a la antibioterapia apoyan la sospecha etiológica. L. monocytogenes es la causa más frecuente de rombencefalitis y en la literatura se ha descrito la afectación clínica y radiológica precoz del nervio trigémino, postulando la hipótesis de que el microorganismo puede tener una diseminación axonal retrógrada al troncoencéfalo a través del nervio trigémino.

**Póster 30**

**Mielitis Longitudinalmente Extensa Seronegativa: Desafíos Diagnósticos y Terapéuticos**

Andrea Irene Cienfuegos Fernández^1^, Julia Pinedo Córdoba^1^, Kevin Jiménez Ureña^1^, María Del Carmen Redondo López^1^, Lourdes Rodríguez-Piñero Moreno^1^, Noelia Guerrero Carmona^1^

^1^Servicio de Neurología, Hospital Universitario Virgen de Valme, Sevilla, España

**Objetivos:** Describir la evolución y el abordaje diagnóstico-terapéutico de una paciente con una mielitis longitudinalmente extensa (MLE) con anti-AQP4 y anti-MOG negativos y posible neuritis óptica (NO) subclínica, con una evolución lenta a pesar del tratamiento. Resaltamos la importancia del seguimiento inmunológico estrecho. **Material y Métodos:** Mujer de 33 años con cervicalgia y parestesias en pies, que progresivamente ascienden hacia región umbilical, acorchamiento en silla de montar y torpeza al caminar. No alteraciones visuales. Balance motor disminuido en MMII que progresa a paraplejia, hipoestesia severa hasta región inframamaria. Hipopalestesia MMII. Parestesias MMSS. Hiperreflexia generalizada. Se realizan PL, RMN cérvico-dorso-lumbar, Body y PET-TC, analítica completa y potenciales evocados visuales (PEV). El tratamiento incluyó corticoides, inmunoglobulinas y plasmaféresis. **Resultados y Conclusiones:** Resultados: RMN cérvico-dorso-lumbar sin contraste y TAC craneal normales. PL: 85 proteínas, 148 leucocitos (77,7% mononucleares). GRAM, cultivo, film-array, PCR virus, ADA negativos. RMN medular con contraste con MLE desde T2, analítica con elevación de VSG. Serología infecciosa negativa. AQP4-IgG, antiMOG-IgG, onconeuronales y marcadores tumorales negativos. ANAs 1/160. Body-TC, PET-TC normales. PEV: alargamiento latencias en ambos ojos. La evolución a pesar del tratamiento fue lenta y con respuesta parcial. Conclusiones: La negatividad para anticuerpos específicos en este caso podría orientar hacia la NMOSD (Trastorno del Espectro de Neuromielitis Óptica) doblemente seronegativa, que representa aproximadamente el 18% de los casos de NMOSD. Sin embargo, el hallazgo de NO subclínica mediante PEV no se contempla en los criterios diagnósticos establecidos, aunque debe tenerse en cuenta. Unos ANAs positivos, aún inespecíficos, obligan a ampliar el estudio y mantener seguimiento estrecho.

**Póster 31**

**Síndrome Confusional Subagudo Secundario a Encefalitis Autoinmune Paraneoplásica por Anticuerpos Anti GABA-B: A Propóstio de un Caso**

Alejandro Molinero Marcos^1^, Ivana Carolina Micheli^1^, Cecilia Coronado Puerto^1^, Manuel García Ortíz^1^, Irene Serrano García^1^, Paola Estefania Pesantes Salinas^1^

^1^Servicio de Neurología, Hospital Universitario Puerta del Mar, Cádiz, España

**Objetivos:** - Exposición del caso de un paciente que debutó con un cuadro confusional subagudo y que, tras estudio etiológico, se diagnosticó de encefalitis autoinmune paraneoplásica. - Resaltar la importancia del diagnóstico y tratamiento precoz, así como del despistaje de lesiones tumorales subyacentes. **Material y Métodos:** Caso clínico. **Resultados y Conclusiones:** Resultados: Varón de 65 años con malestar general, nerviosismo, discurso incoherente y delirios. A la exploración presentó movimientos anormales en extremidades superiores compatibles con mioclonías positivas multifocales. Presentó una crisis epiléptica tónico-clónica, seguida de risa inmotivada, lenguaje incoherente y bloqueos lingüísticos. El paciente fue ingresado para estudio. La analítica mostró hiponatremia leve y reactantes de fase aguda elevados. En líquido cefalorraquídeo (LCR) se objetivó pleocitosis mononuclear y leve hiperproteinorraquia, con FilmArray negativo. El electroencefalograma (EEG) reveló lentificación generalizada; la resonancia magnética cerebral, inflamación de ambos cíngulos. Se detectaron anticuerpos anti-GABA-B en sangre y LCR, iniciándose tratamiento con corticoides e inmunoglobulinas. Se realizó Body-TC con hallazgo de adenopatías mediastínicas, que tras biopsia confirmó tratase de un carcinoma microcítico de pulmón, iniciando tratamiento quimioterápico con buena respuesta clínica. Conclusiones: Este caso refuerza que el diagnóstico precoz es clave, ya que permite no solo un mejor pronóstico con el trata-miento inmunosupresor, cuya efectividad es mayor cuanto más temprano se instaura, sino también la detección temprana de una posible neoplasia subyacente, pudiendo mejorar significativamente la evolución tanto del cuadro inmunomediado como del proceso tumoral asociado.

**Póster 32**

**Síndrome Cordonal Posterior Secundario a Toxicidad por Óxido Nitroso: A Propósito de un Caso**

Alejandro Molinero Marcos^1^, Ivana Carolina Micheli^1^, Cecilia Coronado Puerto^1^, Manuel García Ortíz^1^, Irene Serrano García^1^, Lorena Melero Mora^1^

^1^Servicio de Neurología, Hospital Universitario Puerta del Mar, Cádiz, España

**Objetivos:** - Mecanismo de acción del óxido nitroso y su relación fisiopatológica con el cuadro clínico. - Exposición del caso de un paciente con un síndrome cordonal posterior por consumo de óxido nitroso. - Consideraciones en diagnóstico diferencial de mielopatía en jóvenes. **Material y Métodos:** Caso clínico. **Resultados y Conclusiones:** Resultados: Varón de 47 años con consumo crónico de óxido nitroso (N_2_O), que presenta acorchamiento en miembros inferiores, sensación constrictiva torácica, pérdida de equilibrio e incapacidad para deambular, sin pérdida de fuerza ni alteraciones esfinterianas. A la exploración se objetivó hipoestesia táctil, propioceptiva y palestésica en miembros inferiores, nivel sensitivo en T7, reflejos exaltados y fuerza conservada. Los estudios analíticos, de orina y líquido cefalorraquídeo (LCR) fueron anodinos. Los potenciales somatosensitivos mostraron afectación severa de cordones posteriores. La resonancia magnética evidenció hiperseñal en columnas posteriores desde T5 a T12, confirmando diagnóstico de síndrome cordonal posterior. Se inició trata-miento con vitamina B12, con leve mejoría del cuadro clínico. Conclusiones: El N_2_O es un gas con propiedades anestésicas y analgésicas de uso médico, cuyo consumo recreativo ha aumentado en jóvenes. El uso crónico puede producir toxicidad neurológica al interferir con el metabolismo de la vitamina B12, inactivándola funcionalmente, incluso con niveles séricos normales. Esto genera disfunción medular, debiendo considerarse en el diagnóstico diferencial de mielopatía subaguda en pacientes jóvenes, especialmente con afectación de cordones posteriores. El reconocimiento precoz y la suspensión del consumo, junto con la reposición de vitamina B12, pueden revertir los síntomas y prevenir secuelas neurológicas permanentes.

**Póster 33**

**Crisis Comiciales Clónicas Cervicotoracoabdominales Sintomáticas a Pequeña Lesión Yuxtacortical Encefálica de Etiología Indeterminada**

M. Begoña Gómez González^1^, Begoña Zapata Macías^1^, Míriam Sillero Sánchez^1^, Ana Rodríguez Román^1^, Ana Gómez Roldós^1^

^1^Servicio de Neurología, Hospital Universitario Puerto Real, Puerto Real, España

**Objetivos:** El diagnóstico diferencial de los eventos paroxísticos de movimientos involuntarios es amplio, y la disparidad semiológica, la negatividad de pruebas complementarias y la ausencia de antecedentes pueden predisponernos a atribuirles una etiología incorrecta. **Material y Métodos:** Varón de 38 años, sin antecedentes médicos, en tratamiento con antiagregación y ramipril desde días antes, por episodios polimorfos: 1. Adormecimiento pectoral izquierdo irradiado al miembro superior y seguido de rigidez y paresia, pérdida de conciencia y ansiedad y confusión al despertar; 2. Parestesias hemifaciales izquierdas que se extienden a hemitronco y hemiabdomen, discurso incoherente, recuperación rápida y amnesia parcial; 3. Giro cefálico, parestesias en hemicara, hemitórax/abdomen izquierdo, sacudidas abdominales y flexoextensión axial, con conciencia preservada y petición de ayuda por parte del paciente. Los episodios aumentaron de frecuencia rápidamente (varios al día). **Resultados y Conclusiones:** Exploración, Analíticas, Electroencefalograma, angioRM de troncos supraaórticos, Líquido cefalorraquídeo y TAC corporal fueron normales o anodinos, pero en la RM cerebral se detectó una pequeña lesión yuxtacortical poscentral derecha, con discreto realce heterogéneo de contraste y no aguda. Los episodios desaparecieron al pautar levetiracetam y clonazepam. En sesión clínica conjunta de varios neurólogos, algunos nos inclinamos por la etiología conversiva de los episodios, respaldada por la normalidad del Electroencefalograma y la localización de las clonías. Las características y localización de la lesión radiológica decantaron la decisión hacia instaurar antiepilépticos; la buena respuesta confirmó el diagnóstico de Epilepsia lesional.

**Póster 34**

**Caso Clínico: Trombosis Venosa Cerebral e Hipotensión Intracraneal Espontánea, Una Asociación Infrecuente**

María Luisa Bustos Moreno^1^, Irene Lorite Fuentes^1^, Alba Rodríguez Martín^1^, Esperanza Salinas Íñigo^1^, Sergio Carlos Navarro Conti^1^, Claudia Ruz Aguilera^1^

^1^Servicio de Neurología, Hospital Universitario Reina Sofía, Córdoba, España

**Objetivos:** La trombosis venosa cerebral (TVC) y la hipotensión intracraneal espontánea (HIE) son entidades infrecuentes, que raramente coexisten. La cefalea, síntoma común, puede enmascarar esta asociación y retrasar el diagnóstico. Se presenta un caso de ambas entidades asociada a fuga de líquido cefalorraquídeo (LCR). **Material y Métodos:** Mujer, 54 años, hipertensa y exfumadora, con antecedentes familiares de trombosis venosa profunda y personales de flebitis. Trabaja cargando peso y debutó con crisis epiléptica tónico-clónica, precedida una semana antes por cefalea frontooccipital pulsátil leve, distinta a sus migrañas habituales, sin datos de ortostatismo. Asoció hipoacusia y acúfenos. **Resultados y Conclusiones:** El TC craneal simple mostró una estructura tubular hiperdensa sugestiva de trombosis de vena cortical, confirmándose en estudio con contraste trombosis de la vena cerebral media y vena anastomótica superior izquierdas. Se inició anticoagulación con Enoxaparina y se realizó estudio de trombofilias, que fue negativo. La RM craneal evidenció, además de la trombosis, engrosamiento dural con realce tras contraste. Se sospechó componente de HIE y se amplió estudio con RM espinal que evidenció colección epidural posterior de T2 a T9 compatible con fuga de LCR. Se realizó parche hemático, consiguiendo mejoría clínica. La coexistencia de TVC e HIE representa un reto diagnóstico por su presentación atípica. La sospecha clínica orientada por signos indirectos en neuroimagen permite un abordaje eficaz que puede evitar complicaciones graves. Es fundamental considerarla en pacientes con cefalea atípica y signos neurológicos focales.

**Póster 35**

**Stroke Mimic Como Forma de Presentación de Meningoencefalitis por Listeria Monocytogenes**

Sara Cárdenas Cubero^1^, Emilio Montero Ramírez^1^, Alexandra Rincón Valencia^1^, Coral Conde Velasco^1^, Joaquín Arzalluz Luque^1^

^1^Servicio de Neurología, Hospital Universitario Virgen Macarena, Sevilla, España

**Objetivos:** La Listeria monocytogenes es un patógeno oportunista con elevado neurotropismo, cuya infección puede presentarse con una amplia variedad de síntomas, incluso fatales. La presentación clínica con focalidad neurológica sin signos meníngeos, fiebre ni otros síntomas o signos infecciosos es poco frecuente y supone un reto diagnóstico, pudiendo llegar a presentarse como stroke mimic. **Material y Métodos:** Presentamos un caso de meningoencefalitis por Listeria que debutó como un síndrome hemisférico izquierdo completo de inicio brusco. **Resultados y Conclusiones:** Varón de 63 años valorado en Urgencias por cuadro brusco de alteración del lenguaje y debilidad de extremidades derechas de 10 horas de evolución. A su llegada, se evidencia síndrome hemisférico izquierdo completo, activándose código ictus sin hallazgos en TC multimodal. Durante el ingreso, se objetivan fluctuaciones clínicas y empeoramiento del nivel de conciencia, repitiéndose neuroimagen urgente, sin cambios. Dada la persistencia de síndrome hemisférico izquierdo y la fluctuación del nivel de conciencia, se completa estudio etiológico con punción lumbar, obteniéndose líquido cefalorraquídeo de características inflamatorias y PCR positiva para Listeria. Ante estos hallazgos, se instaura tratamiento antibiótico dirigido con evolución favorable y resolución de síntomas al alta. Se completó también estudio con EEG en vigilia y RM de cráneo, ambos sin hallazgos destacables. La presentación clínica de focalidad neurológica brusca con fluctuaciones posteriores y alteración del nivel de conciencia, nos obliga a replantear el diagnóstico más allá del origen isquémico, especialmente cuando no hay ningún hallazgo en neuroimagen. Una entidad infrecuente, pero de vital importancia es la meningoencefalitis bacteriana por Listeria monocytogenes.

**Póster 36**

**Mielopatía Cervicodorsal Secundaria al Consumo de Óxido Nitroso**

Elena Guijarro Albaladejo^1^, Alexandra Rincón Valencia^1^, Sara Cárdenas Cubero^1^, Ana Sánchez Gómez^1^, Antonio Bocero García^1^, Joaquín Arzalluz Luque^1^

^1^Servicio de Neurología, Hospital Universitario Virgen Macarena, Sevilla, España

**Objetivos:** Los efectos adversos asociados al consumo de óxido nitroso son potencialmente graves y el incremento de su uso recreativo hace que sea necesario conocerlos para identificar su implicación en la creciente incidencia de mielopatía en jóvenes. **Material y Métodos:** Se presenta el caso de un varón de 23 años sin antecedentes que acude a urgencias por un cuadro subagudo de 15 días de evolución de parestesias tetra apendiculares, con empeoramiento progresivo hasta dificultarle la marcha. A la exploración, debilidad 4/5 en la mayoría de los grupos musculares de forma simétrica con REMs hipoactivos. Nivel sensitivo D14, hipopalestesia severa en MMII y leve en MMSS, dismetría generalizada y marcha atáxica. **Resultados y Conclusiones:** Se realiza TC craneal, punción lumbar y ENG-EMG con resultados de normalidad, en RM se visualiza mielopatía cervicodorsal longitudinalmente extensa con predominio de afectación de cordones posteriores. Analíticamente, cifras normales de vitamina B12 con niveles elevados de homocisteína y ácido metilmalónico. Serologías y marcadores de autoinmunidad negativos. Se administra tratamiento con inmunoglobulinas IV sin mejoría. Posteriormente el paciente admite consumo de óxido nitroso, por lo que se suplementa con vitamina B12 con evolución favorable, persistiendo al alta leve marcha atáxica. La afectación medular inducida por óxido nitroso es una patología cada vez más prevalente en la población joven y requiere de un alto índice de sospecha clínica. La manifestación más frecuente es la mielopatía cordonal posterior secundaria al déficit o inactivación funcional de la vitamina B12. La mayoría de los pacientes mejoran con tratamiento sustitutivo, rehabilitación y el abandono del consumo de tóxicos.

**Póster 37**

**Síndrome de Handl con Disfunción Parietal en Spect: Evidencia Funcional en un Diagnóstico Clínico**

Coral Conde Velasco^1^, Emilio Montero Ramírez^1^, Ignacio Ruiz Salcedo^1^, Alexandra Rincón Valencia^1^, Joaquín Arzalluz Luque^1^, Joan Montaner Villalonga^1^

^1^Servicio de Neurología, Hospital Virgen Macarena, Sevilla, España

**Objetivos:** El síndrome de HaNDL (Headache with Neurological Deficits and CSF Lymphocytosis) es una entidad neuro-lógica autolimitada de probable etiología postinfecciosa o inmunomediada, caracterizada por cefalea de perfil migrañoso, déficits neurológicos transitorios y pleocitosis linfocitaria en LCR. Se presenta típicamente en adultos jóvenes sin patología estructural subyacente y cursa con resolución espontánea en semanas. Su diagnóstico es clínico, apoyado en un LCR inflamatorio y la exclusión de causas vasculares, infecciosas o autoinmunes. Se describe un caso representativo con hallazgos funcionales mediante SPECT cerebral. **Material y Métodos:** Mujer de 57 años, con antecedentes de migraña sin aura episódica de baja frecuencia, que consulta por episodios recurrentes de cefalea hemicraneal izquierda asociados a focalidad neurológica transitoria alternante (afasia, hemihipoestesias). A su llegada a urgencias presenta afasia y cefalea, resto de exploración normal. Se realiza TC multimodal urgente sin hallazgos. El LCR muestra pleocitosis linfocitaria (257 células/µL) e hiperproteinorraquia, sin consumo de glucosa. Microbiología, PCR virus (incluyendo WNV) y citología negativa. RM cerebral sin hallazgos. EEG con enlentecimiento delta intermitente anterior izquierdo. SPECT cerebral con hipoperfusión parietal izquierda. Evolución clínica favorable. Tras la introducción de topiramato como preventivo, la paciente se mantiene asintomática. **Resultados y Conclusiones:** La hipoperfusión cortical observada en el SPECT aporta evidencia funcional poco descrita en esta entidad y refuerza su fisiopatología como disfunción cortical reversible de base inflamatoria. Este caso ilustra el valor de técnicas funcionales en entidades clínicamente bien definidas, y subraya la importancia de considerar HaNDL en pacientes con cefalea, focalidad neurológica y LCR inflamatorio, evitando tratamientos innecesarios.

**Póster 38**

**Síndrome de Percheron. Ictus Isquémico Bitalámico. A Propósito de dos Casos con Distinta Evolución Clínica**

Coral Conde Velasco^1^, Álvaro Laviana Martín^1^, Ignacio Ruiz Salcedo^1^, Reyes De Torres Chacón^1^, Soledad Pérez Sánchez^1^, Joan Montaner Villalonga^1^

^1^Servicio de Neurología, Hospital Virgen Macarena, Sevilla, España

**Objetivos:** El tálamo es irrigado por arterias originadas en la base del cráneo, terminales y sin anastomosis funcionales. La arteria de Percheron (AdP) es una variante anatómica en la cual un único vaso, originado en el segmento P1 de una ACP, se encarga de la vascularización bitalámica, con alcance variable al mesencéfalo posibilitando el compromiso de ambos tálamos por afectación unilateral. **Material y Métodos:** Dos pacientes de 67 y 85 años, con FRCV, son traídos a urgencias por bajo nivel de consciencia. El primer paciente estuporoso, con parálisis total de la mirada vertical y hemiparesia izquierda, con lesión isquémica subaguda en región medial de ambos tálamos y mesencéfalo, sin oclusión de gran vaso. Flutter auricular asociado. La segunda paciente comatosa con tetraparesia de predominio derecho. TC con oclusión de segmento P1 de ACP derecha con penumbra isquémica en ambos tálamos. Se detecta FA *de novo*. **Resultados y Conclusiones:** Durante el ingreso, el primer paciente presenta mioclonías corticosubcorticales no epilépticas de origen vascu-lar. La RMN muestra transformación hemorrágica bitalámica y lesiones hiperintensas en difusión hasta núcleos del tercer par, posiblemente por degeneración walleriana. Al alta, recuperación parcial; vigil y con hemiparesia izquierda. La segunda paciente al alta estuporosa y con tetraparesia. La oclusión de la AdP constituye el 0,2% de los ictus isquémicos y su diagnóstico precoz supone un desafío. La alteración del nivel de conciencia, los síntomas oculomotores y los trastornos cognitivoconductuales constituyen la tríada característica. La infrecuencia del cuadro y la baja sensibilidad del TAC inicial explican la dificultad diagnóstica y la importancia de su conocimiento.

**Póster 39**

**Síndrome Amnésico Hipocampal y Biomarcador Positivo Para Enfermedad de Alzheimer: No Obviar la Neuroimagen Estructural**

Antonio Cristóbal Luque Ambrosiani^1^, Carlota Villar Rodríguez^2^, Ernesto García Roldán^1^, Alba Marta Marín Cabañas^2^, María Bernal Sánchez-Arjona^1^, Emilio Franco Macías^2^

^1^Servicio de Neurología, Sevilla, España

^2^Servicio de Neurología, Hospital Universitario Virgen del Rocío, Sevilla, España

**Objetivos:** Describir un caso de síndrome amnésico hipocampal, con biomarcador de enfermedad de Alzheimer (EA) positivo y hallazgo de patología dual grave. **Material y Métodos:** Varón de 68 años; estudios hasta diplomatura. Consulta por pérdida de memoria. Vida independiente. Un año antes había sufrido una caída con TCE, realizándose entonces TC de cráneo. Evaluación actual con exploración neuropsicológica, revisión de TC de cráneo previo y completándose estudio con PET amiloide y, finalmente, RM de cráneo. **Resultados y Conclusiones:** La exploración neuropsicológica muestra MMSE 25/30 (orientación 9/10, atención sostenida 4/5, memoria diferida 0/3). TMA-93 (memoria asociativa visual): 4/30 (1 intrusión). FCSRT+IR, verbal: Recuerdo libre total, 3; Recuerdo Total, 13; Intrusiones, 20. Fluencia verbal semántica, 13. Boston 15-ítems: 10/15. Copia figura simple de RBANS: 19/20. VOSP localización de números: 9/10. FAQ: 4/33. TC de cráneo previo (un año antes, en relación TCE): No lesiones intracraneales. PET amiloide: Positivo, con acúmulo generalizado de amiloide. Se diagnostica EA prodrómica, pero se revisa el TC de cráneo asociado al PET amiloide, visualizándose imagen de LOE temporal izquierdo. RM de cráneo: Imagen de glioblastoma multiforme a nivel hipocampal izquierdo. Se realiza resección quirúrgica, con diagnóstico patológico de glioblastoma IDH, no mutado, grado IV. En la nueva era de uso generalizado de biomarcadores para el diagnóstico de EA, no debe obviarse la neuroimagen estructural, incluso en casos con síndrome amnésico hipocampal típico y biomarcador de EA positivo.

**Póster 40**

**Manifestaciones Neurológicas Como Forma de Presentación de Arteritis de Células Gigantes**

Fernando Francisco Ortega Ortega^1^, Víctor Carmona Bravo^1^, Juan Loscertales Castaños^1^, Joan Montaner Villalonga^1^, Soledad Pérez Sánchez^1^, Ana Sánchez Gómez^1^

^1^Servicio Neurología, Hospital Universitario Virgen Macarena, Sevilla, España

**Objetivos:** La Arteritis de Células Gigantes (ACG) es una vasculitis inflamatoria que afecta a arterias de mediano y gran calibre, como la aorta y sus ramas principales. Sus manifestaciones neurológicas más frecuentes incluyen cefalea, claudicación mandibular y síntomas visuales como la neuritis óptica isquémica arterítica (NOIA), diplopía o amaurosis fugaz (AF), siendo el ictus una presentación infrecuente. **Material y Métodos:** Caso único de hospitalización en planta de Vascular y seguimiento en consultas. **Resultados y Conclusiones:** Varón de 82 años con antecedentes de hipertensión, diabetes tipo 2, fibrilación auricular anticoagulada y ceguera previa en ojo izquierdo por NOIA. Acude a urgencias por visión borrosa, indolora y de inicio brusco en ojo derecho, sin escotomas, con agudeza visual de 0,7. Refiere además cefalea bitemporal leve subaguda. Ante la sospecha de NOIA, se inicia tratamiento con metilprednisolona a altas dosis. Una semana antes fue valorado por un episodio de inestabilidad brusca, objetivándose en TC una lesión isquémica subaguda en el pedúnculo cerebeloso derecho. Las pruebas analíticas mostraron anemia leve, VSG de 22 mm/h y PCR de 17,4 mg/L. El Doppler reveló el signo del halo en arteria temporal izquierda. La angio-TC mostró estenosis en ambas arterias vertebrales, y la RM lesiones isquémicas en territorio posterior. Cumpliendo >6 puntos en los criterios ACR/EULAR 2022 y tras descartar otras causas, se realizó biopsia de arteria temporal, confirmando el diagnóstico de ACG e iniciándose tratamiento con Tocilizumab por Reumatología. El caso destaca la importancia de un diagnóstico precoz en pacientes mayores con comorbilidad vascular, donde la sospecha clínica puede ser baja.

**Póster 41**

**¿Neurología? Sospecho un Código Ictus: La Importancia del Diagnóstico Diferencial en los Imitadores del Ictus, a Propósito de un Caso de Enfermedad de Fahr Como Stroke Mimic**

Jorge Román Rueda^1^, Josefina Andrada Moreno^1^, Carlota Villar Rodríguez^1^, Diego Villagran Sancho^1^, Francisco Hernández Ramos^1^

^1^Servicio de Neurología, Hospital Universitario Virgen del Rocío, Sevilla, España

**Objetivos:** La enfermedad de Fahr es una entidad neurológica infrecuente, caracterizada por calcificaciones cerebrales bilaterales y simétricas en ganglios basales y otras estructuras profundas. Puede presentarse como un stroke mimic, simulando un evento isquémico agudo, lo que dificulta el diagnóstico en contexto de urgencia. **Material y Métodos:** Mujer de 52 años, fumadora activa (30 cigarrillos/día), sin antecedentes neurológicos, psiquiátricos ni familiares relevantes. Acude a urgencias en mayo de 2024 por episodio brusco de disartria, ataxia postural, vómitos y alteración conductual. **Resultados y Conclusiones:** La exploración neurológica evidenció disartria leve e imposibilidad de la marcha por inestabilidad, activándose código ictus. El TC craneal mostró calcificaciones simétricas en núcleos lenticulares y cápsula interna, sin signos de isquemia o hemorragia. Doppler TSA y transcraneal sin alteraciones hemodinámicas. El cuadro se revirtió completamente en menos de 12 horas. La paciente refirió episodios similares previos, autolimitados. RM cerebral sin lesiones estructurales, estudios analíticos, metabólicos, autoinmunes, toxicológicos, cardiológicos y electrofisiológicos fueron normales. A los tres meses permanecía asintomática. La enfermedad de Fahr debe considerarse en el diagnóstico diferencial de síndromes neurológicos agudos atípicos. Aunque clásicamente se asocia a trastornos del movimiento, deterioro cognitivo, crisis epilépticas o síntomas psiquiátricos, puede manifestarse como evento isquémico transitorio. Su reconocimiento permite evitar terapias inadecuadas, como trombólisis innecesaria, y optimizar el manejo mediante un enfoque etiológico estructurado.

**Póster 42**

**Esclerosis Múltiple Remitente Recurrente de Inicio Agresivo (ADEM-LIKE). A Propósito de un Caso**

Irene Serrano García^1^, Ivana Carolina Micheli^1^, Cecilia Coronado Puerto^1^, Lorena Melero Mora^1^, Paola Estefanía Pestantez Salinas^1^, Manuel García Ortíz^1^

^1^Servicio de Neurología, Hospital Universitario Puerta del Mar, Cádiz, España

**Objetivos:** Se presenta el caso clínico de un paciente con esclerosis múltiple remitente-recurrente de inicio agresivo, con una presentación inicial sugestiva de encefalomielitis aguda diseminada (ADEM-LIKE). **Material y Métodos:** Varón de 33 años que debuta con disartria, ataxia, confusión, alteración visual y desorientación. No tenía antecedentes previos de infección. En TAC craneal se objetivan lesiones hipodensas bihemisféricas. Se realiza PL en Urgencias que fue normal (incluido FilmArray, cultivos). La RM informa de posible ADEM sin poder descartar EM con lesiones tumefactivas. Se realiza RM médula que objetiva lesión captante a nivel C3–C4 y datos de atrofia medular. El estudio del LCR muestra bandas oligoclonales positivas, negativas en suero, y serología negativa para anti-AQP4 y anti-MOG. Se inicia tratamiento con corticoides intravenosos y plasmaféresis, obteniendo mejoría parcial y posteriormente con Natalizumab. **Resultados y Conclusiones:** Evolucionó favorablemente, con recuperación motora, desaparición de síntomas cognitivos agudo, marcha cautelosa y mínima afectación sensitiva. El paciente se ha mantenido libre de brotes y sin progresión radiológica. Este caso clínico pone de relieve la dificultad del diagnóstico diferencial entre ADEM y EM de inicio agresivo, especialmente en adultos jóvenes con síntomas encefalopáticos iniciales. Aunque ADEM suele considerarse una entidad monofásica1,2, se han descrito casos con evolución bifásica o progresión a EM, lo que exige un seguimiento estrecho y reevaluación diagnóstica3. ADEM suele estar más relacionado a Ac anti-MOG, más frecuente en niños, pero puede darse en adultos4. Este caso respalda la utilidad de un abordaje multidisciplinar y del tratamiento inmunomodulador precoz como claves para la recuperación funcional y el control de la enfermedad.

**Póster 43**

**Síndrome de Tapia Como Debut de Paraganglioma Yugular: A Propósito de un Caso**

Lourdes Rodríguez-Piñero Moreno^1^, María Del Carmen Redondo López^1^, Julia Pinedo Córdoba^1^, Andrea Cienfuegos Fernández^1^, Kevin Jiménez Ureña^1^

^1^Servicio de Neurología, Hospital Universitario Virgen de Valme, Sevilla, España

**Objetivos:** Los paragangliomas de cabeza y cuello son tumores poco frecuentes que pueden extenderse hacia espacios vasculares o agujeros foraminales, provocando síntomas por compresión de estructuras, como el síndrome de Tapia. **Material y Métodos:** Mujer de 47 años sin antecedentes relevantes que acude a urgencias por alteración progresiva de la movilidad lingual, disfonía y disfagia de varios meses de evolución. En la exploración se observa desviación de la lengua hacia la izquierda, atrofia de hemilengua izquierda y fasciculaciones. Úvula centrada, reflejo nauseoso conserva-do y sin debilidad del esternocleidomastoideo. La fibrolaringoscopia revela parálisis de la cuerda vocal izquierda. Ante la sospecha de una lesión estructural, se realiza resonancia magnética que muestra una masa sólida, p-lilobulada, en el golfo de la yugular izquierda, con afectación del canal hipogloso y la pared posterior del canal carotídeo. La imagen presenta vacíos de señal, compatibles con la apariencia típica en “sal y pimienta”. **Resultados y Conclusiones:** La exploración neurológica es compatible con afectación de los pares craneales X y XII. La afectación simultánea de ambos define el síndrome de Tapia. Las características radiológicas y la expansión anatómica orientaron al diagnóstico de paraganglioma yugular. Ante un cuadro clínico compatible con el síndrome de Tapia, sin antecedentes de intubación orotraqueal, debe considerarse una etiología tumoral. Al tratarse de una causa potencialmente grave pero tratable, siendo el paraganglioma yugular el tumor más frecuente en esta localización.

**Póster 44**

**Microdeleción del Cromosoma 17q21. Importancia del Estudio Genético en Pacientes Crisis Epilépticas y Fenotipo Característico**

Francisco Javier Serrano García^1^, Jerónimo cruces Párraga^1^, Pablo Doblas Ráez^1^, Claudia Rúz Aguilera^1^, Jose Carlos Estévez María^1^

^1^Servicio de Neurología, Hospital Universitario Reina Sofia, Córdoba, España

**Objetivos:** Las alteraciones cromosómicas comprenden un amplio grupo de enfermedades y síndromes con diversas manifestaciones clínicas. Podemos encontrar entre ellas al Síndrome de Koolen-de Vries, provocado por la microdeleción del cromosoma 17q21 la cual induce cambios alterando el gen KANSL1. Hasta un 33% de los pacientes con este síndrome padecen crisis epilépticas a lo largo de su vida. **Material y Métodos:** En esta serie de casos, presentamos a una mujer joven de 24 años con facies sindrómica, tics cefálicos, conductas obsesivas o estereotípicas, retraso madurativo y crisis focales motoras de miembro superior derecho, así como generalizadas, con difícil control pese a tratamiento con fármacos anticrisis desde el inicio en la infancia. **Resultados y Conclusiones:** La paciente ha sido estudiada desde la infancia tanto por Neurología pediátrica como por neurología de adul-tos. Durante su estudio en consultas, debido a que presentaba un fenotipo característico y retraso madurativo, se cursaron análisis genéticos, objetivándose una microdeleción del cromosoma 17q21. Tras haber empleado varios fármacos anticrisis sin una adecuada respuesta, bien por bajo control de la frecuencia de las crisis o por intolerancia/efectos secundarios. Actualmente, se encuentra en tratamiento con Lacosamida 100-0-150, con un control adecuado sin presentar crisis desde la instauración de la pauta. Con este caso, pretendemos remarcar la importancia del estudio genético en pacientes con crisis epilépticas criptogenéticas así como una adecuada exploración y reconocimiento temprano que pueden ayudar a reconocer este tipo de síndromes genéticos.

**Póster 45**

**Síndrome Confusional/Episodio Psicótico con Focalidad Neurológica en Mujer Transexual Bajo Tratamiento con Testosterona**

María Begoña Gómez González^1^, Míriam Sillero Sánchez^1^, Ana Rodríguez Román^1^, Ana Gómez Roldós^1^, Beatriz Rosado Peña^1^, Lucía Moreno Vico^1^

^1^Servicio de Neurología, Hospital Universitario Puerto Real, Puerto Real, España

**Objetivos:** La hormonoterapia se ha relacionado con síntomas y diversas enfermedades neurológicas, cuyo ejemplo más reconocido es la migraña. El uso de tratamiento hormonal se ha incrementado actualmente como parte del trata-miento de las personas transexuales. La testosterona puede exacerbar los síntomas de trastornos psiquiátricos ya conocidos, y es menos probable que desencadene trastorno conductual en pacientes sin tales antecedentes. **Material y Métodos:** Mujer transexual de 21 años tratada con testosterona, que presentaba alteración de la percepción (sombras y voces hostiles), diplopia ocasional, cefalea, insomnio y dificultad para controlar los impulsos. Carecía de antecedentes psiquiátricos. **Resultados y Conclusiones:** Fue hospitalizada, y RM craneal con angioRM venosa, Ecografía carotídea, Electroencefalograma, Serología, Líquido cefalorraquídeo, Anticuerpos antineuronales, resultaron normales, así como la exploración. Se trató durante su estancia con benzodiacepinas y al alta se remitió a Psiquiatría y a Endocrinología para ree-valuación de la terapia hormonal. En el paciente joven es más frecuente la aparición de una enfermedad psiquiátrica. La terapia hormonal puede actuar como desencadenante o ser el único factor causal; en cualquier caso, el diagnóstico debe realizarse habiendo excluido otras etiologías. Tras el alta, nuestra paciente fue atendida en el Servicio de Urgencias por sobreingesta medicamentosa, y actualmente, sin tratamiento hormonal, se ha recuperado.

**Póster 46**

**Encefalitis Autoinmune Anti-Hu y Anti-CASPR2: A Propósito de un Caso**

Antonio Guzmán Téllez^1^, Ana Campos Villegas^1^, Ester Morales García^1^, Leah Rivas Ramos^1^, Carlos Segura Sanz^1^

^1^Servicio de Neurología, Hospital Universitario Virgen de la Victoria, Málaga, España

**Objetivos:** Describir un caso clínico de una paciente con encefalitis autoinmune anti-Hu y anti-CASPR2 positiva, con mala evolución. **Material y Métodos:** Reporte de caso clínico. **Resultados y Conclusiones:** Paciente de 68 años sin antecedentes de interés ingresada por cuadro confusional subagudo con agitación y deterioro progresivo del nivel de consciencia que inició 5 días antes de acudir a urgencias. El líquido cefalorraquídeo mostró hiperproteinorraquia y monocitosis sin consumo de glucosa. Presentó un empeoramiento clínico que desembocó en ingreso en unidad de cuidados críticos con necesidad de ventilación mecánica. Se completó el estudio con los siguientes hallazgos: un grado severo de afectación cerebral difusa en el electroencefalograma junto a engrosamiento paquimeníngeo difuso en resonancia con focos de restricción en difusión a nivel frontal y parietal derecho. Positividad para anticuerpos anti-Hu en suero y anti-CASPR2 en suero y líquido cefalorraquídeo. Dada la implicación de estos anticuerpos se realizó un estudio de despistaje tumoral en el que solo se evidenció en la tomografía por emisión de positrones (PET) un nódulo pulmonar hipercaptante planteándose su intervención que no llegó a realizarse por el fallecimiento de la paciente. Recibió tratamiento con corticoterapia, inmunoglobulinas, plasmaféresis, rituximab y ciclofosfamida sin mejoría. Este caso nos ilustra un ejemplo de encefalitis de alta sospecha paraneoplásica en las que el tratamiento debe ser precoz y pese a ello, el pronóstico es complejo. Es primordial el despistaje neoplásico para enfocar un correcto tratamiento tumoral que permita dilucidar un mejor desenlace.

**Póster 47**

**Del Arco Aórtico al Tronco Cerebral: Ictus Isquémico por Aortitis Autoinmune**

Macarena González Campos^1^, Gonzalo Torres Sánchez^1^, Hayar El Mouhajir Mohamed^1^, Gemma Sanz Fernández^1^

^1^Servicio de Neurología, Hospital Juan Ramón Jiménez, Huelva, España

**Objetivos:** La aortitis es una inflamación de la pared aórtica que puede tener etiología infecciosa o autoinmune. Puede ser una causa subyacente de ictus isquémico, especialmente en el territorio posterior. Exponemos un caso sobre un ictus isquémico vertebrobasilar secundario a vasculitis. **Material y Métodos:** Varón de 62 años, sin antecedentes, que debutó bruscamente con diaforesis, mareo, hemiparesia izquierda y descenso de consciencia. Se activó código ictus, a su llegada se objetivó oftalmoparesia, parálisis facial izquierda, tetraparesia con mejoría espontánea a hemiparesia izquierda, disartria leve y dismetría derecha (NIHSS 8). En neuroimágen se visualizaba imagen lineal en arteria basilar sugerente de disección. Se inició fibrinolísis, dada la mejoría con NIHSS 1 se optó por actitud conservadora. La RMN/angioRMN descartó disección. Durante el estudio, se objetivó en ETT y ETE dilatación y engrosamiento de la raíz aórtica, realizando diagnóstico diferencial entre hematoma mural vs. origen inflamatorio, por lo que se amplió con doppler de arterias temporales, marcadores analíticos de inflamación y PET-TAC, evidenciándose vasculitis de aorta ascendente y cayado aórtico. Inició corticoterapia con evolución asintomática. **Resultados y Conclusiones:** La aortitis por vasculitis de gran vaso explica <1% de los ictus. La inflamación parietal induce trombosis mural y embolias proximales que imitan disección. Cuando TC-RM es inconcluyente, la combinación de ecocardiografía y PET TAC muestra captación circunferencial, confirma vasculitis y orienta inmunosupresión precoz, reduciendo recurrencia isquémica. Aunque infrecuente, la aortitis puede originar ictus vertebro-basilar; debe considerarse ante infartos posteriores criptogénicos acompañados de hallazgos aórticos. Reconocerla permite iniciar inmunosupresión temprana y evitar recurrencias isquémicas.

**Póster 48**

**Stroke Mimic por Masa Paravertebral: Desafíos Diagnósticos y Enfoque Multidisciplinario**

Ana Campos Villegas, Carmen Ortega Hiraldo, Trinidad Sanjuán Pérez

Servicio de Neurología, Hospital Universitario Virgen de la Victoria, Málaga, España

**Objetivos:** Una proporción significativa de pacientes inicialmente orientados como ictus isquémico presentan finalmente diagnósticos alternativos como tumores del SNC, epilepsia focal con estado postcrítico, trastornos metabólicos o mielopatías compresivas entre otros diagnósticos diferenciales. **Material y Métodos:** Descripción de un caso clínico **Resultados y Conclusiones:** Varón de 67 años, fumador de un paquete de tabaco al día como antecedentes. Comienza con clínica aguda de debilidad y alteración sensitiva de miembro superior derecho. Tras no mejoría en días, acude a urgencias. Tras exploración clínica se objetiva debilidad 4/5 en extensión carpo, flexión dedos e hipoestesia comparativa en mano derecha. Se cursa ingreso en Neurología orientándose el caso como ictus isquémico subagudo de perfil lacunar sensitivo motor. Durante ingreso se realiza RM craneal donde se objetiva gran masa paravertebral derecha con destrucción de apófisis transversas C1–C4, con estenosis severa de canal C2–C3. El paciente se remite para estudio por parte de Otorrinolaringología, sin ser posible la toma de muestras pese a la realización de dos biopsias. Durante el ingreso, episodios de dificultad respiratoria por efecto masa de la lesión, con necesidad de traqueostomía El paciente pasa a cargo de Neurocirugía, donde finalmente se resecciona parcialmente la tumoración. La anatomía patológica describe la lesión compatible con una malformación vascular venosa y linfática con afectación de hueso y de partes blandas. Este caso destaca la importancia de considerar diagnósticos alternativos como son las lesiones compresivas atípicas, en el diagnóstico diferencial de ictus, subrayando la necesidad de un enfoque multidisciplinario en estos pacientes.

**Póster 49**

**Hemorragia Intracerebral Tras Uso de Sildenafilo**

Bernardo Cueli Rincón^1^, Ana Rodríguez Reguera^2^, María Jesús Rivera-Pinna García^1^, María Del Carmen Robles Román^2^, María Elena Calzado Rivas^1^, Francisco Javier De La Torre Laviana^2^

^1^Unidad de Gestión Clínica de Neurología y Neurofisiología, Jerez De La Frontera, España

^2^Servicio de Neurología, Hospital Universitario de Jerez de la Frontera, Jerez De La Frontera, España

**Objetivos:** Sildenafilo es un fármaco inhibidor de la 5-fosfodiesterasa con efecto vasodilatador, con indicación principal en la disfunción eréctil, siendo seguro con supervisión facultativa, aunque con aparición de eventos adversos a nivel cardiológico y vasculocerebral. Describimos a un paciente que desarrolló un ictus hemorrágico tras la toma de Sildenafilo. **Material y Métodos:** Paciente de 60 años, antecedentes de hipertensión arterial sin tratamiento, enolismo moderado y poliartritis mi-crocristalina hiperuricémica, mRankin basal de 0, que presentó a finales de 2024, un cuadro clínico brusco nueve horas evolucionado de disartria, paresia faciohemicorporal derecha leve-moderada de predominio braquial, tras la ingesta de Sildenafilo adquirido sin prescripción, con cifras discretamente elevadas de la presión arterial; leve mejoría de sintomatología motora días posteriores, con desarrollo de poliartritis inflamatoria microcristalina que mejoró con tratamiento corticoideo, con mRankin de 3 al alta. **Resultados y Conclusiones:** La TC craneal inicial detectó una hemorragia aguda en el núcleo lenticular y brazo posterior de la cápsula interna izquierdos, con AngioTAC cerebral sin alteraciones relevantes, monitorización en la Unidad de Ictus, sin constatar arritmias embolígenas, con control de la presión arterial, estudios analíticos a destacar una leucocitosis de predominio neutrofílico inicial, aumento de los reactantes de fase aguda, con artrocentesis que no apreció datos sépticos. La correcta prescripción de Sidenafinilo, junto con el adecuado control de los factores de riesgo vascular evitaría la aparición de efectos adversos importantes. El manejo inicial del ictus hemorrágico en la Unidad de Ictus puede mejorar el pronóstico funcional del mismo. La detección precoz de la sintomatología ictal debe seguir implementándose a nivel poblacional.

**Póster 50**

**Vértigo Posicional Paroxístico Central Como Manifestación Principal de Ictus Isquémico de la Arteria Cerebelosa Anteroinferior**

Ana Sánchez Gómez^1^, Álvaro Laviana Marín^1^, Joaquin Arzalluz Luque^1^, Antonio Bocero García^1^, Soledad Pérez Sánchez^1^, Joan Montaner Villalonga^1^

^1^Servicio de Neurología, Hospital Universitario Virgen Macarena, Sevilla, España

**Objetivos:** El ictus de la arteria cerebelosa anteroinferior (AICA) es una entidad clínica poco frecuente que afecta a pedúnculo cerebeloso medio, cerebelo y protuberancia lateral. Sus principales manifestaciones son vértigo, ataxia, parálisis facial e hipoacusia, con alta correlación clínicoradiológica. **Material y Métodos:** Mujer de 78 años, hipertensa, diabética y con hipoacusia bilateral, que acude a Urgencias por síndrome vertiginoso de inicio brusco de una semana de evolución, desencadenado exclusivamente con cambios posturales y giros cefálicos, con inicio brusco y empeoramiento escalonado hasta impedir la deambulación. **Resultados y Conclusiones:** A la exploración, se observa leve nistagmo horizontorrotatorio intermitente hacia la izquierda sin otro nistagmo posicional, dismetría apendicular izquierda, hipoestesia en territorios V2/V3 izquierdos y empeoramiento de hipoacusia basal en oído izquierdo, NIHSS de 3 puntos. En TAC de cráneo se objetiva una hipodensidad en pedúnculo cerebeloso medio izquierdo. Se completa estudio con RMN, que muestra la lesión hipointensa en T1 e hiperintensa en T2/FLAIR, con restricción a la difusión y sin captación de contraste; y angio-TAC con estenosis crítica de segmento V4 derecho y afectación arteriosclerótica de la arteria basilar. Finalmente se diagnostica de ictus en territorio de AICA izquierda, de etiología aterotrombótica y posible mecanismo embólico arterioarterial. Es dada de alta con doble antiagregación. El ictus de la AICA es poco frecuente y puede confundirse con trastornos vestibulares periféricos. La combinación de vértigo, hipoacusia y ataxia sugiere afectación troncoencefálica y cerebelosa. La presencia de síntomas cocleares puede ayudar a diferenciarlo de otros síndromes vestibulares y de fosa posterior.

**Póster 51**

**Síndrome de Encefalopatía Posterior Reversible Como Debut de Angiopatía Amiloide Inflamatoria Cerebral. Descripción de un Caso**

Alejandro Molinero Marcos^1^, Ivana Carolina Micheli^1^, Cecilia Coronado Puerto^1^, Manuel García Ortíz^1^, Irene Serrano García^1^, Ana Gómez Roldós^2^

^1^Servicio de Neurología, Hospital Universitario Puerta del Mar, Cádiz, España

^2^Servicio de Neurología, Hospital Universitario Puerto Real, Cádiz, España

**Objetivos:** - Exponer el caso clínico de un paciente con alteración del estado mental a causa de una angiopatía amiloide inflamatoria cerebral que cursó clínicamente con un síndrome PRES-like. - Consideraciones en el diagnóstico diferencial clínico-radiológicas entre ambas patologías. **Material y Métodos:** Caso clínico. **Resultados y Conclusiones:** Resultados: Varón de 83 años con inicio agudo de encefalopatía, crisis epilépticas y cefalea en contexto de hipertensión arterial mal controlada. Se completó estudio con RM cerebral que mostró hiperintensidades en sustancia blanca parietooccipital bilateral compatibles con síndrome de encefalopatía posterior reversible (PRES), y lesiones microhemorrágicas adyacentes multifocales que cumplían criterios de Boston; un electroencefalograma (EEG) que mostró una encefalopatía moderada, y análisis de LCR que reveló pleocitosis e hiperproteinorraquia leves, con descenso de péptidos β-amiloide 40 y 42. Se inició tratamiento con metilprednisolona en bolos y nimodipino, con recuperación clínica completa. Conclusiones: La angiopatía amiloide inflamatoria cerebral (AAI) es una entidad rara, caracterizada por una respuesta inflamatoria frente a depósitos de β-amiloide en vasos leptomeníngeos y corticales. Suele presentarse con deterioro cognitivo-conductual subagudo, aunque también se han descrito cefalea, crisis epilépticas y déficits neurológicos focales. Es preciso tenerla en cuenta en el diagnóstico diferencial en pacientes con cuadros clínico-radiológicos sugestivos de PRES por sus similitudes clínico-radiológicas. La excelente respuesta a corticoides, la presencia de microhemorragias cercanas a las zonas hiperintensas en RM cerebral y la alteración del amiloide en LCR apoyan el diagnóstico de AAI. Además, es importante reseñar las implicaciones pronósticas que conllevan el diagnóstico y el tratamiento inmunosupresor precoces.

**Póster 52**

**Síndrome Piramidal Progresivo y Hemiatrofia Cerebral de Inicio en la Edad Adulta con Positividad de Bandas Oligoclonales: Encefalopatía Crónica Tipo Rasmussen**

María Begoña Gómez González^1^, Ana Gómez Roldós^1^, Alicia Sánchez Refolio^1^, Beatriz Rosado Peña^1^, Ana Rodríguez Román^1^

^1^Servicio de Neurología, Hospital Universitario Puerto Real, Puerto Real, España

**Objetivos:** El síndrome hemisférico izquierdo progresivo de inicio en la edad adulta requiere un estudio amplio por las múltiples posibilidades etiológicas. La encefalitis de Rasmussen predomina en la infancia/adolescencia, y su diagnóstico requiere presencia de crisis epilépticas, síntomas progresivos y atrofia radiológica hemisférica. Cuando aparece en el adulto y no dominan la sintomatología las crisis epilépticas, el proceso diagnóstico se prolonga y requiere la exclusión de otras enfermedades. **Material y Métodos:** Varón de 46 años remitido por el Servicio de Rehabilitación (junio 2023) por debilidad, parestesias y temblor intencional en la mano izquierda. La RM craneal y cervical mostró una pequeña protrusión discal y probable gliosis frontoparietal derecha. Como Antecedentes, constaban Fibrilación auricular permanente, obesidad y tabaquismo. En la Exploración, había discreto hipobraceo izquierdo y discretas bradicinesia, dismetría y debilidad en la mano izquierda, signo de Babinski en el pie izquierdo y atrofia en el adductor del pulgar. **Resultados y Conclusiones:** Se practicaron Electromiograma (normalidad), SPECT cerebral (sin degeneración nigroestriatal), nueva RM craneal (hemiatrofia cerebral derecha severa frontal y parietal), Electroencefalograma (brotes de puntas y ondas lentas generalizadas) y estudio de Líquido cefalorraquídeo (bandas oligoclonales, positivas en líquido y negativas en suero), determinación de Anticuerpos Antineuronales/Serología/Metales pesados (negativa). A pesar de su inicio tardío y no haberse identificado crisis epilépticas clínicas, y después de haberse excluido razonablemente parkinsonismo degenerativo, patología de motoneurona o mielopatía, se estableció el diagnóstico de probable Encefalitis de Rasmussen.

**Póster 53**

**Respuesta a Cannabidiol en Paciente con Síndrome de Rett y Epilepsia Refractaria Asociada**

Lorena Melero Mora^1^, Benito Galeano Bilbao^1^, Cecilia Coronado Puerto^1^, Manuel García Ortíz^1^, Irene Serrano García^1^, Paola Pesantez Salinas^1^

^1^Servicio de Neurología, Hospital Universitario Puerta del Mar, Cádiz, España

**Objetivos:** El síndrome de Rett es un trastorno grave del neurodesarrollo ligado al gen MECP2. Cursa con múltiples manifestaciones neurológicas, entre ellas epilepsia de difícil control. Presentamos el caso de una paciente con cuadro compatible con epilepsia refractaria y alteración del neurodesarrollo con óptima respuesta a cannabidiol en el contexto de síndrome de Rett. **Material y Métodos:** Paciente de 21 años con mutación presente en el gen MECP2, que en la primera infancia debuta con epilepsia refractaria a tratamiento y regresión del desarrollo compatible con encefalopatía epiléptica y del neurodesarrollo (EEND). Desde los 7 años presenta cuadros sugestivos de crisis comiciales que se manifiestan como automatismos inicial y posteriormente como crisis tónico-clónicas generalizadas y focales con alteración del nivel de conciencia, las cuales no se autolimitan tras tratamiento a dosis plenas con ácido valproico, lamotrigina, topiramato y clobazam en monoterapia o combinadas. **Resultados y Conclusiones:** Paciente con 7–10 crisis diarias en politerapia, que tras el inicio de cannabidiol a dosis de 4 mL cada 12 horas asociado a topiramato, lamotrigina y clobazam consigue una reducción significativa de las crisis con adecuada tolerancia. El uso de cannabidiol fuera de sus indicaciones habituales puede ser una opción terapéutica prometedora para el tratamiento de epilepsias refractarias en el contexto de síndromes genéticos, como es el síndrome de Rett.

**Póster 54**

**Plexopatía Isquémica Bilateral: Un Caso Clínico Poco Frecuente**

Hayar El Mouhajir Mohamed^1^, Gonzalo Torres Sánchez^1^, Macarena Gonzalez Campos^1^, Gemma Sanz Fernández^1^

^1^Servicio de Neurología, Hospital Juan Ramon Jiménez, Huelva, España

**Objetivos:** La plexopatía isquémica bilateral es una condición de presentación infrecuente, que se caracteriza por la disfunción de los plexos nerviosos, ya sea braquial o lumbosacro, como consecuencia de un compromiso vascular. Esta patología resulta especialmente relevante debido a su rareza y a la complejidad clínica asociada con su diagnóstico y manejo, ya que puede ocasionar discapacidades importantes si no se trata. **Material y Métodos:** Presentamos el caso de una mujer de 35 años que ingresa a Urgencias por dolor lumbar y pérdida de fuerza en miembros inferiores, en el contexto de una trombosis aórtica aguda. **Resultados y Conclusiones:** • Trombosis terminal aórtica aguda que ocupa casi toda la aorta abdominal infrarrenal distal y ambas arterias ilíacas comunes. • Embolectomía transfemoral bilateral de urgencia, con amputación infracondílea en miembro inferior derecho. • Infarto esplénico extenso evidenciado en angio-TC. • Plexopatía lumbosacra de origen isquémico probable tras la trombosis aórtica, posiblemente asociada a un hematoma en el músculo ilíaco izquierdo (PET-TC). • Electroneurografía: afectación extensa del plexo lumbosacro izquierdo, con denervación aguda y ausencia de actividad voluntaria. • Alteraciones medulares en RM sugestivas de hiperplasia medular/reconversión hematopoyética. • Anemia microcítica e hipocrómica asociada a ferropenia. • Estudio autoinmune y de trombofilias primarias negativo, aunque con anticuerpos anticardiolipina positivos a títulos no diagnósticos. La escasez de literatura sobre esta entidad subraya la importancia de su diagnóstico temprano y un manejo integral. Este caso destaca la necesidad de un seguimiento multidisciplinario para rehabilitación funcional, manejo del dolor, optimización hematológica y prevención de eventos trombóticos recurrentes, asegurando una mejor calidad de vida para la paciente.

**Póster 55**

**Distonía Aguda Paroxística Como Manifestación de Brote de Esclerosis Múltiple por Lesión Desmielinizante en Ganglios Basales**

Adelaida Cuenca Relinque^1^, Juan Luis Ruiz Peña^1^, Fernando Francisco Ortega Ortega^1^, Sara Cárdenas Cubero^1^, Marta Calle Serrano^1^

^1^Servicio de Neurología, Hospital Universitario Virgen Macarena, Sevilla, España

**Objetivos:** Presentar un caso de distonía focal paroxística aguda como manifestación inicial de un brote de esclerosis múltiple recurrente – remitente (EMRR) secundaria a una lesión desmielinizante en ganglios basales, y discutir la fisiopatología subyacente. **Material y Métodos:** Paciente con EMRR que presentó un cuadro de distonía focal paroxística aguda sintomático a una lesión desmielinizante localizada en ganglios basales. Se procede a revisión de la RMN con contraste, análisis del EEG y evaluación de la respuesta al tratamiento con carbamazepina. **Resultados y Conclusiones:** Mujer de 41 años, con EMRR diagnosticada en 2013, que presentó episodios paroxísticos breves de contracción involuntaria tónica de hemicara derecha y cuello, que se repetían varias veces al día. La RMN mostró nueva lesión hiperintensa en T2 FLAIR y DWI en la región capsulo talámica izquierda, con realce periférico de contraste en anillo incompleto, sugestiva de lesión desmielinizante aguda. El EEG fue normal. La distonía remitió completamente tras el inicio de carbamazepina. La distonía en este contexto se atribuye a la disfunción de los ganglios basales, estructuras cruciales en la regulación del movimiento, secundaria a la lesión desmielinizante aguda. La interrupción de las vías inhibitorias y excitatorias dentro de los ganglios basales puede desencadenar patrones de contracción muscular anormales y sostenidos. La resolución con carbamazepina sugiere un componente de hiperexcitabilidad neuronal. La distonía focal paroxística aguda puede ser una manifestación inicial de un brote de EMRR asociada a lesiones desmielinizantes en ganglios basales. El reconocimiento de esta presentación clínica y la comprensión de su fisiopatología son cruciales para un diagnóstico y tratamiento oportunos.

**Póster 56**

**Cefalea Ortostática y Fuga: Descripción de un Caso de Hipotensión Licuoral**

Alejandro Morillas Pinteño^1^, Martina Violeta Jiménez Alonso^1^, Carmen Mercado Begara^1^, Patricia Gil Armada^1^

^1^Servicio de Neurología, Hospital Universitario de Jaén, Jaén, España

**Objetivos:** Describir un caso de hipotensión de LCR espontáneo (SIH: Spontaneous Intracranial Hypotension). La semiología asociada a este cuadro suele causar una cefalea ortostática que mejora con el decúbito y que no suele responder de forma significativa a la analgesia habitual. **Material y Métodos:** Se ha realizado una revisión de la literatura de hipotensión de LCR espontánea a través de Pubmed, Scopus y Google Scholar. Se presentan las características clínicas y los hallazgos radiológicos de una paciente con este síndrome, comparándolos con la evidencia científica reciente. **Resultados y Conclusiones:** - Resultados: Mujer de 40 años que consulta en Urgencias en varias ocasiones debido a cefalalgia de un mes de evolución sin desencadenante evidente ni signos compatibles con rinolicuorrea u otolicuorrea. Las características de esta cefalea son: opresiva, holocroneal, asociando náuseas y vómitos. Refiere empeorar con el ortostatismo y mejo-rar con el decúbito, pero no con analgésicos. No refiere síntomas ni signos sugestivos de focalidad neurológica. Asimismo, ha presentado dos episodios de pérdida de consciencia sin relajación de esfínteres, ni mordida lingual, ni movimientos involuntarios. - Discusión: Es fundamental conocer los datos clínicos que pueden llevar al diagnóstico de SIH, ya que actualmente es una patología infradiagnosticada e infratratada. Por ello, debemos considerar la posibilidad de este síndrome, incluso cuando no se reporta una punción lumbar reciente y se evidencian signos de cefalea ortostática. No obstante, la ausencia de este síntoma no descarta el diagnóstico, ya que la presentación clínica puede variar considerablemente y puede hacer que el diagnóstico sea desafiante.

